# Large-scale discovery of non-conventional peptides in grape (*Vitis vinifera* L.) through peptidogenomics

**DOI:** 10.1093/hr/uhac023

**Published:** 2022-05-02

**Authors:** Mao-Song Pei, Hai-Nan Liu, Tong-Lu Wei, Yi-He Yu, Da-Long Guo

**Affiliations:** College of Horticulture and Plant Protection, Henan University of Science and Technology, Luoyang, 471023, Henan Province, China; Henan Engineering Technology Research Center of Quality Regulation and Controlling of Horticultural Plants, Luoyang 471023, China; College of Horticulture and Plant Protection, Henan University of Science and Technology, Luoyang, 471023, Henan Province, China; Henan Engineering Technology Research Center of Quality Regulation and Controlling of Horticultural Plants, Luoyang 471023, China; College of Horticulture and Plant Protection, Henan University of Science and Technology, Luoyang, 471023, Henan Province, China; Henan Engineering Technology Research Center of Quality Regulation and Controlling of Horticultural Plants, Luoyang 471023, China; College of Horticulture and Plant Protection, Henan University of Science and Technology, Luoyang, 471023, Henan Province, China; Henan Engineering Technology Research Center of Quality Regulation and Controlling of Horticultural Plants, Luoyang 471023, China; College of Horticulture and Plant Protection, Henan University of Science and Technology, Luoyang, 471023, Henan Province, China; Henan Engineering Technology Research Center of Quality Regulation and Controlling of Horticultural Plants, Luoyang 471023, China

## Abstract

Non-conventional peptides (NCPs), which are peptides derived from previously unannotated coding sequences, play important biological roles in plants. In this study, we used peptidogenomic methods that integrated mass spectrometry (MS) peptidomics and a six-frame translation database to extensively identify NCPs in grape. In total, 188 and 2021 non-redundant peptides from the *Arabidopsis thaliana* and *Vitis vinifera* L. protein database at Ensembl/URGI and an individualized peptidogenomic database were identified. Unlike conventional peptides, these NCPs derived mainly from intergenic, intronic, upstream ORF, 5′UTR, 3′UTR, and downstream ORF regions. These results show that unannotated regions are translated more broadly than we thought. We also found that most NCPs were derived from regions related to phenotypic variations, LTR retrotransposons, and domestication selection, indicating that the NCPs have an important function in complex biological processes. We also found that the NCPs were developmentally specific and had transient and specific functions in grape berry development. In summary, our study is the first to extensively identify NCPs in grape. It demonstrated that there was a large amount of translation in the genome. These results lay a foundation for studying the functions of NCPs and also provide a reference for the discovery of new functional genes in grape.

## Introduction

Small peptides are defined as peptides with 2–100 amino acids, and they play an important role in diverse biological processes [1]. For instance, the discovery and application of insulin have completely changed the quality of life of diabetic patients. Small signal peptides in plants, such as the cysteine-rich peptides LAT52, LeSTIG1, and MAPK, participate in self-incompatibility responses [2]. In the past few years, research on small peptides has focused mainly on conventional peptides (CPs) derived from annotated coding sequences [1]. Recently, a new type of endogenous peptide, characterized as non-conventional peptides (NCPs), has gradually attracted the interest of researchers. NCPs are peptides derived from previously unannotated coding sequence (CDS) regions, including intergenic regions, introns, untranslated regions (UTRs), etc.

Although NCPs are derived from unannotated coding regions, more and more studies have shown that NCPs play important biological roles in plants. For example, POLARIS participates in the modulation of *Arabidopsis thaliana* root growth and leaf vascular patterning [3]. ROTUNDIFOLIA4 regulates *A. thaliana* leaf shape [4], and OSIPs are involved in oxidative stress tolerance of *A. thaliana* [5]. Moreover, some NCPs derived from hairpin-containing primary transcripts (pri-miRNAs) have received significant attention for functionally promoting the expression of corresponding miRNAs. For instance, miPEP171d, miPEP171b, and miPEP165a are reported to regulate root development by promoting their corresponding mature miRNAs [6–7]. miPEP164c inhibits proanthocyanin synthesis and stimulates anthocyanin accumulation in grape berry cells [8]. All these studies indicate that NCPs are indispensable for the development of plants. However, because of the short fragments of these peptides, they are usually ignored in gene prediction and mass spectrometry (MS) analysis, which leads to serious underestimation of the total number and diversity of plant peptides.

With the increasing importance of NCPs, their identification has received more and more attention. The emergence of high-throughput sequencing technology makes it possible to identify NCPs on a large scale. Computational approaches based on sequence similarity through cross-species comparisons are one of the methods used to identify NCPs [9]. However, because NCPs are generally short, the computational approach is not very effective owing to low conservation scores. Some researchers use other methods such as ribosome profiling (Ribo-seq) to identify NCPs [10–11]. Ribo-seq is a high-throughput sequencing technology that provides information about genome-wide transcripts that are being translated. This approach relies on the ability of translation ribosomes to protect RNA fragments of 20–30 nucleotides from nuclease digestion. Another new method, peptidogenomics, integrates MS peptidomics and genomics and is gradually becoming the main method for identifying NCPs [1,12].

Although some researchers have identified peptides through multiple methods, as in *Arabidopsis* and maize [1,12–13], the demonstration of the biological functions of NCPs remains. NCPs function by modulating larger regulatory proteins, and their functions can therefore be predicted from the proteins on which they act [14]. In addition, the functions of NCPs can also be predicted by genome-wide association studies, such as the combination of NCPs with quantitative trait locus (QTL) or domestication analysis [1]. Studies on the NCPs of grape berries are scarce, and only a few studies on peptides encoded by primary miRNA sequences have been reported [6]. All such studies have shown that the NCPs have important functions and cannot be ignored.

In this study, we collected berries of three developmental stages from “Kyoho” based on the EL system [15]. Total protein was then extracted and filtered through a 10-kDa ultrafiltration tube. After peptide desalination, MS and chromatography experiments were performed. To identify more NCPs, we used a combination of standard and customized databases. The standard database was retrieved from the Ensembl protein databases of grape and *A. thaliana.* The customized database was constructed based on a six-frame translation. PEAKS Studio software was used to search the MS results against the two databases mentioned above. In total, we identified 188 and 2021 non-redundant peptides from the *A. thaliana* and *Vitis vinifera* L. protein database at Ensembl/URGI and the individualized peptidogenomic database, respectively. Then the chromosome distributions of NCPs and CPs, as well as the origins of NCPs, were analyzed. The results indicated that the NCPs were widespread in the grape genome, and the distribution patterns of NCPs and CPs on chromosomes were different. To analyze the functions of NCPs, we also compared the locations of NCPs with those of QTLs, LTR retrotransposons, and domestication selection regions. About 94% of the NCPs were in QTLs, such as those associated with development, intrinsic quality, disease resistance, and fruit quality. We also investigated whether the NCPs showed developmental specificity. The large-scale NCPs identified here provide important information for our understanding of these small molecules in grape.

## Results

### Peptidogenomic process for NCP identification

**Figure 1:**
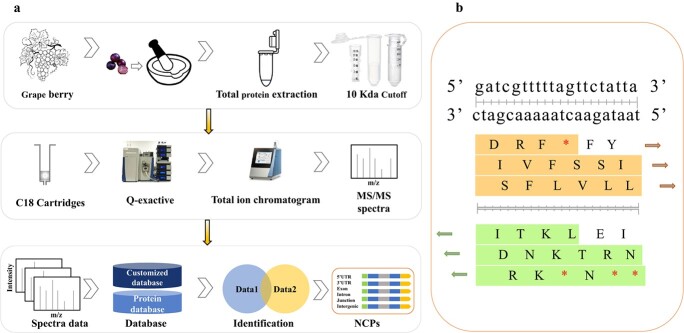
Flow chart of grape NCP identification. **a:** The workflow of grape NCP identification. The endogenous peptides were extracted using conventional methods. Then the peptides were enriched from the total grape berry proteins through a 10-kDa cutoff filter, and C18 cartridges were used to desalt the peptide mixtures. The polypeptides were separated by capillary HPLC and analyzed by MS with a Q Exactive Plus mass spectrometer. PEAKS Studio was used to search against the URGI protein database for grape, the Ensembl protein database for Arabidopsis, and the individualized peptide database to identify peptides. **b:** Construction of customized grape peptidogenomic database. The sixpack function of emboss-6.6.0 [16] was used to construct the potential peptide database. Each peptide terminated at the stop codon, and the next peptide then started after the previous stop codon. The location information of each putative peptide was recorded and stored in FASTA format.

To detect the NCPs in grape berries, we used a peptidogenomic process as shown in Fig. 1a. The total proteins of grape berries were extracted according to the experimental method. Then a 10-kDa cutoff filter was used to enrich the peptides from the total proteins, and C18 cartridges were used to desalt the peptide mixtures. To obtain endogenous peptides widely present in grapes, PEAKS Studio was used to search the resulting mass spectrum dataset against the *A. thaliana* and *V. vinifera* L. protein database at Ensembl/URGI and an individualized peptidogenomic database. The construction of the individualized peptidogenomic database was based on a six-frame translation of the grape genome sequences (Fig. 1b). Accordingly, a 4.07 × 10^9^ (B) individualized peptidogenomic database (45 664 424 sequences) was obtained.

### Identification and distribution of CPs and NCPs in grape

**Figure 2:**
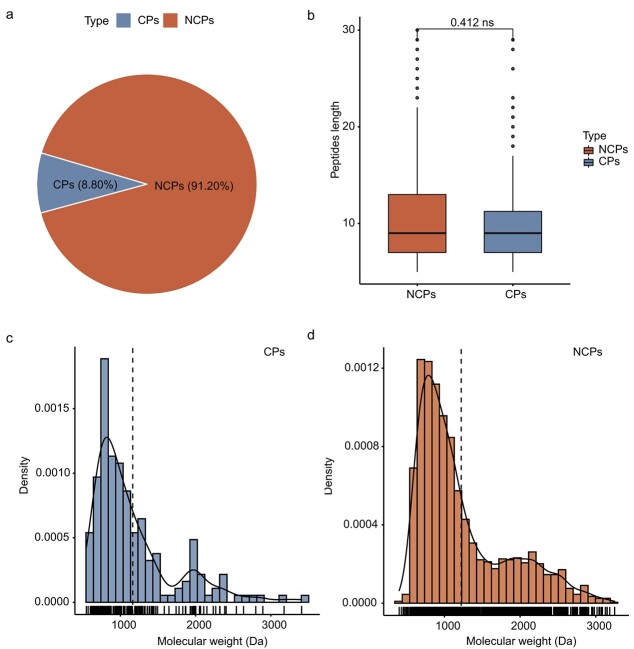
Overview of peptide identification in grape. **a:** Pie chart showing numbers of CPs and NCPs identified. **b:** Boxplot of length distribution of CPs and NCPs. Hypothesis testing with Wilcox test (^*^p < 0.05). **c:** Statistics for CP molecular weights (188). **d:** Statistics for NCP molecular weights (2021).

In total, we identified 188 and 2021 non-repetitive peptides from the grape-*Arabidopsis* Ensembl/URGI protein database and the individualized peptidogenomic database, respectively (Table S1 and S2). Of these, 1897 NCPs and 183 CPs were assigned to a single genomic locus (Fig. 2a; Tables S3 and S4). The median lengths of CPs and NCPs were 10.1 and 10.6, and there was no significant difference in length between CPs and NCPs (Fig. 2b). About 90% of the CPs and NCPs contained fewer than 17 and 20 amino acids, respectively. The average molecular weight (AMV) of the NCPs was 1220.87 Da, and peptides with molecular weights less than 2500 Da accounted for 95.20% (1925). The AMV of the CPs was 1163.376 Da, and 96.80% (182) of these peptides had a molecular weight less than 2500 Da (Fig. 2c and 2d). These results indicated that NCPs are an important component of plant proteins.

**Figure 3:**
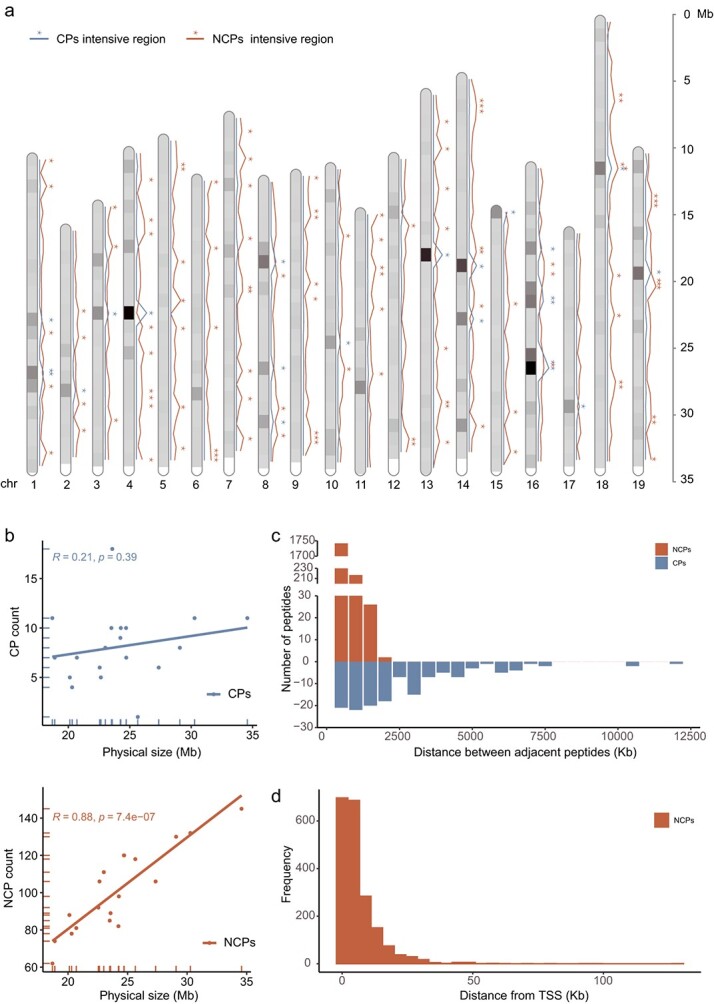
Distribution of CPs and NCPs in grape. **a:** Distribution statistics for grape genome CPs and NCPs. The blue line represents CPs, and the orange line represents NCPs. The ^*^ represents hotspot regions (window size = 1 Mb). **b:** Correlations between CP and NCP counts and physical size with the Levenberg–Marquardt (LM) method. **c:** Distance distribution of two neighboring CPs or NCPs. **d:** Histogram of the distances from each NCP and CP to the neighboring TSS.

The distribution of both NCPs and CPs on grape chromosomes was uneven (Fig. 3a), and 124 intensive regions (defined by 1-Mb windows) were identified (Fig. 3a). Among these, 22 CPs of intensive regions containing 28 peptides (18.18%) were obtained, whereas 102 NCPs of intensive regions containing 721 peptides (37.61%) were identified (Fig. 3a). Among these intensive regions, four regions located on chr1, chr16, chr18, and chr19 were shared by both CPs and NCPs. There was no correlation between the number of CPs and chromosome length (R = 0.21, *p* = 0.39), but the number of NCPs and chromosome length were correlated (R = 0.88, *p* = 7.4e−07) (Fig. 3b).

To estimate the coverage of peptides over the genome, the intervals between adjacent peptides (kb) were calculated. The results showed that 87.67% (1742) of the NCPs were within 500 kb of each other, whereas only 14.89% [21] of the CPs were within 500 kb of each other (Fig. 3c). Moreover, 32.14% (645) of the NCPs were located within 2 kb of the adjacent canonical translation start site (TSS) (Fig. 3d). These results indicated that NCPs were widespread in the grape genome, and the distribution patterns of NCPs and CPs on chromosomes were different.

Previous studies have shown that most endogenous peptides start with non-AUG codons [1, 17]. In this study, the mRNA sequences of NCPs and CPs revealed that most NCPs and CPs had non-AUG TSSs (Table S3 and Table S4). Although it is well known that the translation initiation site in eukaryotes is AUG, our results revealed that non-AUG-initiated translation is also widespread. These results were consistent with MS analysis [18].

### Analysis of NCPs originating from coding or non-coding regions

**Figure 4:**
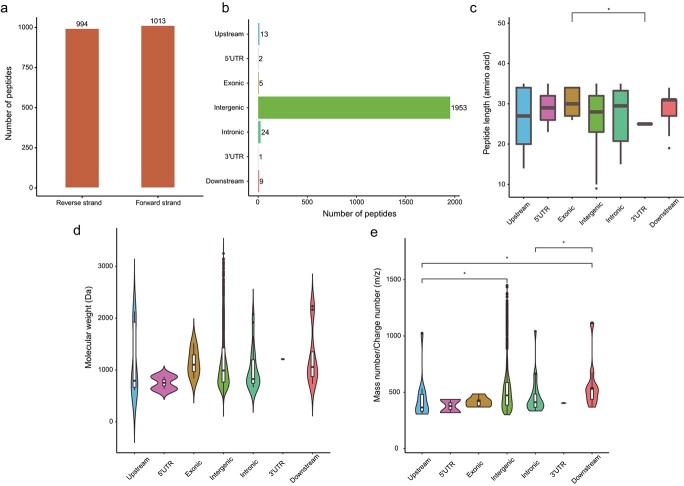
Features of grape NCPs. **a:** Histogram of the number of NCPs on sense and antisense strands. **b:** Numbers of NCPs that originated from different regions. **c–e:** Length, molecular weight, and mass number/charge number ratio of NCPs from different regions. Hypothesis testing with Wilcox test (^*^p < 0.05).

An analysis of NCP origins showed that 49.5% (994) were located on the reverse strand in grape (Fig. 4a). A gene resource analysis showed that 1953 (97.31%) of the NCPs originated from intergenic regions, 24 (1.20%) from intronic regions, 13 (0.65%) from upstream regions (defined as 1 kb from the TSS), 9 (0.45%) from downstream regions, 5 (0.25%) from exonic regions, 2 (0.10%) from 5′UTR regions, and 1 (0.050%) from a 3′UTR region (Fig. 4b). These results suggest that non-coding sequences were translated. Length analysis indicated that NCPs derived from exonic regions were longer than that derived from the 3′UTR region (Fig. 4c). Analysis of molecular weights showed that 70% of the NCPs were less than 1300 Da, and the AMV of NCPs from upstream, 5′UTR, exonic, intergenic, intronic, 3′UTR, and downstream regions did not differ significantly (Fig. 4d and Table S5). The mass number/charge number (m/z) ratio of NCPs derived from upstream and intergenic regions, upstream and downstream regions, and intronic and downstream regions were significantly different (Fig. 4e).

To verify the NCPs identified in this study, the NCPs were compared with published long non-coding RNA (lncRNA) sequencing data from grape [19]. The results showed that 22 NCPs were derived from lncRNAs (Table S6).

### Verification of NCPs

**Figure 5:**
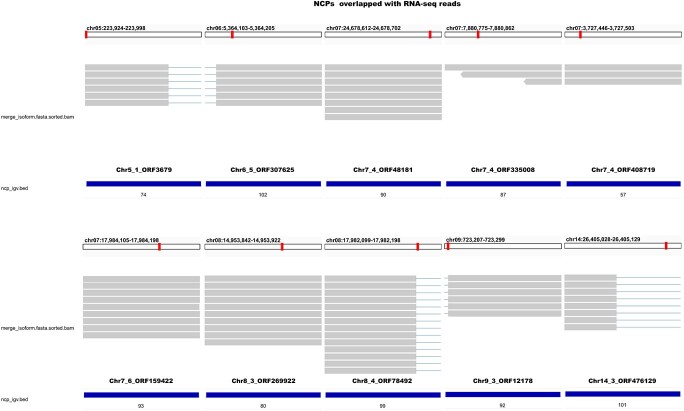
The overlap of NCPs with RNA-seq reads. The overlap of NCP locations with PacBio SMRT Iso-Seq data from “Kyoho” grape berries is shown. The red box represents the position of NCPs on the chromosome.

To determine whether NCPs from non-conventional regions were transcribed, we investigated the overlap of NCP locations with PacBio SMRT Iso-Seq data from “Kyoho” grape berries deposited in the Sequence Read Archive (PRJNA790655) (https://dataview.ncbi.nlm.nih.gov/object/PRJNA790655). The results showed that 87 NCPs overlapped with the RNA-seq reads (Table S7). Then 10 NCPs were randomly selected for display with IGV [20] (Fig. 5). These results indicated that the NCPs were indeed transcribed.

### NCPs located in regions associated with QTLs

**Figure 6:**
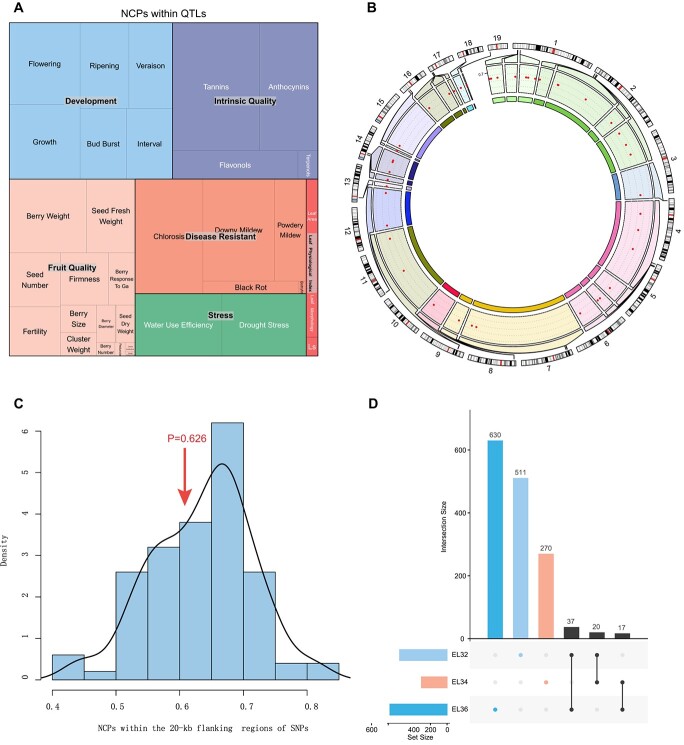
QTLs and selection regions associated with grape NCPs and comparison of developmental periods. **a:** NCPs associated with QTLs. **b:** NCPs that overlap with selection regions. The red dots indicate the zoom sectors of NCPs located in selection regions. **c:** NCPs within 20-kb flanking regions of SNPs. The “pnorm” function in R was used to calculate the upper-tail test *p*-values (lower.tail = FALSE). **d:** Comparison of NCPs in different developmental periods.

In grape, many quantitative trait loci (QTLs) associated with various traits have been identified [21]; these include QTLs related to fruit quality, stress, disease resistance, intrinsic quality, and leaf physiological index (Table S8). The relationship between QTLs and NCPs was investigated, and the results showed that most NCPs (94%) were located in QTLs (Fig. 6a; Table S9). Among the QTLs related to development, most NCPs were in regions related to “flowering” and “growth”. In the intrinsic quality category, the top two terms were “tannins” and “anthocyanins”. In the disease resistance category, “chlorosis” and “downy mildew” were the dominant terms. In the fruit quality category, “berry weight” and “seed fresh weight” were the main terms. There were also many NCPs located in regions with “water use efficiency”, “drought stress”, and “leaf area”. These results showed that NCPs may play a potential role in the regulation of plant growth, development, and resistance.

Long terminal repeat retrotransposons (LTR-RTs) are ubiquitous and dominant repetitive elements in plant genomes and play a crucial role in genomic diversity, phenotypic variation, and evolution [22]. All the LTR-RTs were identified using LTR_retriever (Table S10). Then the relationship between NCPs and LTR-RTs was investigated, and 47 NCPs were identified in LTR-RT regions (Table S11). The functions of these NCPs remain to be explored.

### The distribution of NCPs in regions associated with selection and phenotypic variation

Grapes, one of the earliest domesticated fruit crops, are widely cultivated for both fresh consumption and winemaking. During grape domestication, wild species were genetically selected for traits that were beneficial to humans, promoting the use of grape as a source of food or material; these traits included flower sex, berry size, sugar content, and berry color. Here, we investigated the relationship between NCPs and domestication, including selective sweeps for domesticated table and wine grapes, wild and domesticated grapes, berry edibility, and stress resistance [23–25]. The results showed that 42 NCPs overlapped with domestication intervals (Fig. 6c, Table S12). However, how NCPs affect domestication traits and the nature of their mechanism of action require further study.

Because of the existence of linked inheritance, the relationship between the 10-kb interval upstream and downstream of SNPs related to investigated traits and NCPs was also investigated [23–24, 26]. In total, 61 NCPs overlapped with the 10-kb intervals upstream and downstream of SNPs related to investigated traits, including berry shape, berry weight, berry color, flower sex, intrinsic quality, etc. (Table S13). Compared to randomly generated genome fragments of the same length and the same number on the same chromosome (see Methods), the plant trait-associated SNPs were not significantly enriched in NCP regions (*p* < 0.626, upper-tail test; Fig. 6c). These results differed from the pattern of NCPs in maize [1], which may indicate that NCPs in different species have unique characteristics.

### NCPs show developmental specificity in “Kyoho” grape

To explore whether the NCPs showed developmental specificity, all NCPs derived from the EL32, EL34, and EL36 developmental stages, which represent “beginning of bunch closure”, “berries begin to soften”, and “berries with intermediate brix values”, respectively, were compared [15]. There were 511, 270, and 630 NCPs derived from EL32, EL34, and EL36, respectively (Fig. 6d). Among these NCPs, 20 were shared by EL32 and EL34, 37 were shared by EL32 and EL36, and 17 were shared by EL34 and EL36 (Table S14). We can therefore conclude that the NCPs showed developmental specificity.

## Discussion

MS is an efficient technique for peptide detection in proteomics research. The standard operating procedure is to map the data from the MS experiment to the annotated genes. MS differs from other techniques such as ribosome profiling and can directly verify the translation of transcripts [14]. Interestingly, to date, there are still a large number of MS fragmentation spectra that have not been identified, mainly because some of the spectra belong to unannotated peptides in proteomics studies. Peptidogenomics is a practical method for identifying unannotated peptides that combines proteomics with the six-frame translation of a genome [27–28]. An individualized database constructed by six-frame translation at a genome-wide scale can be used to effectively identify all possible peptides [29–30]. Although peptidogenomics has been successfully used to identify peptides in microorganisms and humans, it has rarely been applied to plants other than maize [1,12,31–33]. In this study, peptidomics and an individualized peptidogenomic database constructed by six-frame translation of the URGI/ENsembl protein databases of grape and *Arabidopsis* were combined. As far as we know, this is the first published work on the peptidogenomic analysis of NCPs in grape. A total of 1897 NCPs and 183 CPs were identified in the customized database and the URGI/ENsembl protein databases. This study demonstrated that the integration of MS and peptidogenomics was an effective method for detecting CPs and NCPs. In our results, most peptides were NCPs, indicating that sequences previously thought to be untranslated, such as 5′UTRs, intergenic regions, introns, and 3′UTRs, are capable of translation.

Recently, more and more studies have shown that the proteins encoded by lncRNAs play an important role in cellular processes. For example, the putative lncRNA-encoded peptide HOXB-AS3 could suppress colon cancer growth [34]. A peptide encoded by an lncRNA could enhance SERCA activity in muscle by activating the calcium pump [35]. The peptide encoded by the putative lncRNA HBVPTPAP was reported to induce apoptosis of hepatocellular carcinoma cells by regulating JAK/STAT signaling pathways [36]. In addition, four lncRNA-encoded peptides identified in moss were shown to regulate growth and differentiation by overexpression and inhibitory expression experiments [37]. In our study, 22 NCPs were found to be derived from lncRNAs in grape (Table S6). Although the functions of these NCPs were unclear, this result lays a foundation for further study of these small peptides encoded by lncRNAs.

Upstream ORF peptides have attracted attention because of their ability to control the translation of downstream ORFs [38–40]. For example, an upstream ORF was reported to repress the expression of a tomato NAC domain transcription factor in a peptide sequence-dependent manner [41]. Upstream ORF-mediated translation of WNK8 is essential for the *Arabidopsis* response to ABA [42]. In this study, 13 NCPs were derived from upstream regions in grape, and 2 NCPs were derived from 5′UTRs. Compared with those from the 5′UTR, peptides derived from the 3′UTR region have traditionally been overlooked. Recently, peptides derived from 3′UTR regions were identified in moss [37]. In this study, we identified only one NCP derived from a 3′UTR, but its biological function should not be ignored.

Although many QTLs have been identified in grape, few studies have paid attention to the relationship between QTLs and NCPs. In maize, studies have shown that QTLs are highly associated with NCPs. Clark *et al*. (2006) [43] showed that the intergenic sequences of maize had a pleiotropic effect on morphology. Castelletti *et al*. (2014) [44] found that NCPs located in the *Vgt1* QTL for maize flowering time were associated with the methylation state. Wang *et al*. (2020) [1] also reported that NCPs were significantly enriched in plant trait-associated QTL regions. Therefore, associating NCPs with QTLs is an effective method for studying their function. In our study, we found that most NCPs (94%) were located in QTLs for traits such as development, intrinsic quality, fruit quality, disease resistance, stress, and leaf physiology. These results indicate that NCPs have an important function in regulating these traits. LTR-RTs are believed to be crucial for maintaining chromatin structure and centromere function, as well as regulating gene expression in the host genome [45]. In this study, LTR-RTs were identified, and the relationship between NCPs and LTR-RTs was analyzed. The functions of NCPs located in LTR-RT regions require further exploration. Domestication is widespread in cultivated crops, and screening for domestication-related genes is very helpful for understanding evolution, which will also advance the domestication of crops. In this study, 42 NCPs overlapped with regions under selection, indicating the underlying functional sites for the evolution of grape.

By comparing the overlap of NCPs at different developmental stages, we found that no peptides were continuously expressed throughout the development of grape berries. This result indicated that NCPs may have specific, time-limited functions in different biological processes. This result is also consistent with the fact that most peptides function as signaling molecules in biological processes. For example, the cysteine-rich peptides LAT52, LeSTIG1, and MAPK participate in self-incompatibility responses [2].

This is the first study to use a peptidogenomic approach to identify NCPs on a large scale in the grape genome. The results indicate that regions previously considered to be untranslated, such as intergenic regions, introns, upstream ORFs, 5′UTRs, 3′UTRs, and downstream ORFs, are in fact translated and have potential functions in biological processes. These findings also provide a reference for the discovery of new functional genes in grape.

## Materials and methods

### Plant materials

Three “Kyoho” grapevines of similar vigor were selected in spring 2021 at the farm of Henan University of Science & Technology, Luoyang, China. The grape berries were harvested at the EL32, EL34, and EL36 stages according to the EL system [15], flash-frozen with liquid nitrogen, and stored at −80°C.

### Peptide extraction

Grape berry samples were weighed (3.0 g) and ground to powder at a low temperature. Then the tissue powder was transferred to an EP tube with 8 M urea lysate and sonicated for 6 min (ultrasonic power 20%, 2 s on/3 s off) on ice to break the tissue. Tubes were centrifuged for 15 minutes at 12000 rpm and 4°C; the supernatant was transferred to a 10-kDa ultrafiltration tube for ultrafiltration separation and centrifuged at 11000 rpm and 4°C.

### Peptide desalination

First, a C18 membrane-packed column was prepared and activated by centrifuging three times with 40 μL methanol. Second, the column was balanced with 40 μL Nano-HPLC Buffer A three times. The dried polypeptide extracts were re-dissolved in Nano-HPLC Buffer A and centrifuged three times on the balanced C18 column. Then, the C18 column was centrifuged three times with 40 μL Nano-HPLC Buffer A for desalting. Finally, the C18 column was centrifuged twice with 40 μL elution phase Buffer B to collect the desalinated polypeptides.

### LC–MS/MS analysis

The dried polypeptide extracts were re-dissolved in Nano-HPLC Buffer A and separated by the Nano-HPLC liquid system on an UltiMate 3000 RSLCnano (Thermo Fisher Scientific, MA, USA). Solution A was 0.1% formic acid-water, and solution B was 0.1% formic acid-acetonitrile. The trap column was balanced with 100% solution A at 3 μL/min (RP-C18, Agilent). Then the samples were loaded by an automatic sampler, combined with the trap column, and separated on an analysis column at a flow rate of 300 nL/min on a 75 μm × 150 mm column (RP-C18, New Objective, USA). Peptides were separated by capillary high-performance liquid chromatography, and MS was performed with a Q Exactive Plus mass spectrometer (Thermo Fisher Scientific). The detection methods were as follows: after calibration with the standard calibration solution, the mother solution was scanned using data dependent acquisition (DDA) mode (350–2000 m/z). Then the 20 strongest fragment profiles (MS2 scan) were collected after high energy collision dissociation (NCE energy 28, dynamic exclusion time 25 s). We set the resolution of MS1 to 70 000 at M/Z 200, the AGC target to 3e6, and the maximum injection time to 100 ms. We also set the resolution of MS2 to 17 500, the AGC target to 1e5, and the maximum injection time to 50 ms.

### Construction of the peptide database

The genomes of grape [46] and *Arabidopsis* [47] were downloaded from URGI (https://urgi.versailles.inra.fr/files/Vini/Vitis%2012X.2%20annotations/12Xv2_grapevine_genome_assembly.fa.zip) and Ensembl (http://ftp.ensemblgenomes.org/pub/plants/release-51/fasta/arabidopsis_thaliana/dna/). The sixpack function of emboss-6.6.0 [16] was used to construct the potential peptide database. Each peptide terminated at the stop codon, and the next peptide was then started after the previous stop codon. The location information of each putative peptide was recorded and stored in FASTA format.

### Peptide identification with PEAK Studio

PEAK Studio v5.3 (Bioinformatics Solutions, Inc.) was used to search against the URGI protein database for grape [46] (https://urgi.versailles.inra.fr/files/Vini/Vitis%2012X.2%20annotations/vitviv2.pep.fasta.zip), the Ensembl protein database for *Arabidopsis* [47] (http://ftp.ensemblgenomes.org/pub/plants/release-51/fasta/arabidopsis_thaliana/pep/), and the individualized peptide database to recognize peptides. The type of peptide-producing sequence was determined using bedtools-v2.25.0 [48]. Peptides derived from annotated CDSs were defined as CPs. Peptides from intergenic regions, UTR regions, reading frames different from those of annotated CDSs, and intronic regions were defined as NCPs.

### Peptide distribution on chromosomes

Peptide density on chromosomes was calculated with the R package RIdeogram::genomicDensity [49] using a sliding window size of 1e6. The distance between peptides and adjacent TSSs was calculated with the Python script (https://github.com/Peims/Calculate-the-distance-between-peptide-and-adjacent-stop-codon) based on the grape genome annotation [46] (https://urgi.versailles.inra.fr/files/Vini/Vitis%2012X.2%20annotations/Vitis_vinifera_gene_annotation_on_V2_20.gff3.zip). Then the distances between peptides and adjacent TSSs were used to draw a frequency plot.

### Verification of NCPs

PacBio SMRT Iso-Seq data from “Kyoho” grape berries deposited in the Sequence Read Archive (PRJNA790655) (https://dataview.ncbi.nlm.nih.gov/object/PRJNA790655) were downloaded, and bedtools-v2.25.0 [48] was used to calculate the overlap between NCPs and RNA-seq reads. The overlap of NCPs with RNA-seq reads was demonstrated using IGV-2.11.9 [20].

### Association of NCPs with QTLs, LTR retrotransposons, and domestication selection

QTLs related to 34 traits, including development, intrinsic quality, fruit quality, disease resistance, stress, and leaf physiological index, were collected (Table S8) [21]. LTR retrotransposons were detected and retrieved from the grape genome [46] (https://urgi.versailles.inra.fr/files/Vini/Vitis%2012X.2%20annotations/12Xv2_grapevine_genome_assembly.fa.zip) using LTRharvest [50] and LTR_Finder [51] (Table S10). The domestication intervals of grape fruits were collected from both wild and domesticated grapevines [23–25]. The NCPs that intersected with the QTLs, LTR retrotransposons, and domestication intervals were selected as candidate NCPs.

### Association analysis of NCPs with SNPs

SNPs were collected from both wild European and domesticated grapevines [23–24, 26]. One hundred genomic sequences were randomly generated as background; each random sequence had the same characteristics as the NCPs, including total quantity, distribution modes on different chromosomes, and peptide length distribution [1] (Fig. S1). The mean and SD of the normal distribution of NCPs were calculated using the 100 random genomic sequences within 20-kb regions flanking SNPs. The “pnorm” function in R was used to calculate the upper-tail test p-values (lower.tail = FALSE); the p-value represented the probability that the observed value exceeded the expected distribution.

## Supplementary Material

Web_Material_uhac023

## Data Availability

LC–MS/MS data from “Kyoho” grape berries at EL32, EL34, and EL36 were deposited in the iProX (integrated proteome resources) (https://www.iprox.org/) database under accession number IPX0003909000 (https://www.iprox.cn/page/project.html?id=IPX0003909000). The release date is 2022-12-29 00:00:00. Data sets produced in the current study and/or analysis may be provided by the corresponding author upon reasonable request.
