# Supplementary material for: Large-scale discovery of non-conventional peptides in grape (Vitis vinifera L.) through peptidogenomics
Source: Hortic Res. 2022 May 2;9:uhac023. doi: 10.1093/hr/uhac023 (PMC9070638; doi:10.1093/hr/uhac023)
Supplement: Web_Material_uhac023 [file web_material_uhac023.zip › manuscript_marked-up.docx]

**Large-scale discovery of** **non-conventional peptides in grape (*Vitis vinifera* L.) through peptidogenomic**

**Running title:** Large-scale discovery of NCPs in grape

**Mao-Song Pei^1,2^, Hai-Nan Liu^1,2^, Tong-Lu Wei^1,2^, Yi-He Yu^1,2^, Da-Long Guo^1,2^***

^1^College of Horticulture and Plant Protection, Henan University of Science and Technology, Luoyang, 471023, Henan Province, China.

^2^Henan Engineering Technology Research Center of Quality Regulation and Controlling of Horticultural Plants, Luoyang 471023, China.

**Email addresses:**

Mao-Song Pei

E-mail: [peimaosong@163.com](mailto:peimaosong@163.com)

Hai-Nan Liu

E-mail: [liuhainan0995@163.com](mailto:liuhainan0995@163.com)

Tong-Lu Wei

E-mail: [9906070@haust.edu.cn](mailto:9906070@haust.edu.cn)

Yi-He Yu

E-mail: [yuyihe@haust.edu.cn](mailto:yuyihe@haust.edu.cn)

Da-Long Guo*

Corresponding author E-mail: [guodalong@haust.edu.cn](mailto:guodalong@haust.edu.cn)

*Correspondence: Da-Long Guo, College of Horticulture and Plant Protection, Henan University of Science and Technology, Luoyang, 471023, Henan Province, China.

Henan Engineering Technology Research Center of Quality Regulation and Controlling of Horticultural Plants, Luoyang 471023, China.

**Abstract**

Non-conventional peptides (NCPs), which refer to peptides derived from previous unannotated coding sequences, play important biological roles in plants. In the study, we adopted a peptidogenomic methods which integrated mass spectrometry (MS) peptidomics and six-frame translation database to extensively identify NCPs in grape. In total, 188 and 2,021 non redundant peptides from *Arabidopsis thaliana* and *Vitis vinifera* L. protein database in Ensembl/URGI and individualized peptidogenomic database were identified. Differ from conventional peptides, these NCPs mostly derived from intergenic, intronic, upstream ORFs, 5’UTR, 3’UTR and downstream ORFs regions. The results showed that the unannotated regions are translated broadly than we thought. We also found that most NCPs were derived from regions related to phenotypic variations, LTR retrotransposons and domestication selection, indicating that the NCPs play an important function in complex biological processes. Moreover, we also found that the NCPs were developmental specificity, and played transient and certain function in grape berry development. In summary, our study was the first time to extensively identify NCPs in grape. And proved that there was a large amount of translation in the genome. The results laid the foundation for studying the function of NCPs and also provided some reference for discovering some new functional genes in grape.

**Key words:** Peptides; NCPs; Peptidogenomic; Grape.

**Introduction**

Small peptides are defined as peptides with 2-100 amino acids, which play an important role in diverse biological processes^1^. For instance, the discovery and application of insulin has completely changed the quality of life of diabetic patients. Small signal peptides in plants, such as cystein-rich peptides LAT52, LeSTIG1 and MAPK participate in self-incompatibility responses^2^. In the past few years, researches on small peptides are mostly about conventional peptides (CPs) derived from the annotated coding sequences^1^. Recently, a new type of endogenous peptides which characterised as non-conventional peptides (NCPs) has gradually attracted the interests of researchers. The NCPs refer to peptides derived from previous unannotated coding sequences (CDSs) regions which including intergenic regions, introns, and untranslated regions (UTRs), etc.

Although NCPs derived from unannotated coding regions, more and more studies showed that NCPs play important biological roles in plants. Such as POLARIS participated in modulating *Arabidopsis* *thaliana* root growth and leaf vascular patterning^3^. ROTUNDIFOLIA4 regulated *Arabidopsis thaliana* leaf shape^4^. OSIPs involved in oxidative stress tolerance of *Arabidopsis thaliana*^5^. Moreover, some NCPs derived from hairpin-containing primary transcripts (pri-miRNAs) has caught significant attention as functionally promoting the expression of corresponding miRNA. For instance, miPEP171d, miPEP171b and miPEP165a were reported to regulate root development by promoting their corresponding mature miRNAs^6-7^. miPEP164c inhibited proanthocyanin synthesis and stimulates anthocyanin accumulation in grape berry cells^8^. All these studies indicated that NCPs are indispensable in the development of plants. However, due to the short fragments of peptides, they are usually ignored in gene prediction and mass spectrometry analysis, which leads to serious underestimation of the total number and diversity of peptides in plants.

With the increasing importance of NCPs, the identification of NCPs has received more and more attention. The emergence of high-throughput sequencing technology makes it possible to identify NCPs on a large scale. Computational approaches based on sequence similarity through cross-species comparisons is one of the methods to identify NCPs^9^. However, because NCPs are generally short, the computational approach is not very effective due to low conservation scores. Some researchers use other methods such as ribosome profiling (Ribo-Seq) to identify NCPs^10-11^. Ribo-seq is a high-throughput sequencing technology which provides information about genome-wide transcripts being translated. This approach relies on the ability of translation ribosomes to protect RNA fragments of 20-30 nucleotides from nuclease digestion. In addition, another new method referred as peptidogenomics that integrated mass spectrometry (MS) peptidomics and genomics is gradually becoming the main method for identifying NCPs^1,12^.

Although some researchers have identified peptides through multiple methods, such as in *Arabidopsis* and maize^1,12-13^. The demonstration of the biological function of NCPs remains. As reported, NCPs function by modulating larger regulatory proteins, hence, their function can be predicated by the protein which they act on^14^. In addition, the function of the NCPs can also be predicated by genome-wide association studies, such as the combination of NCPs and quantitative trait locus (QTL) or domestication analysis^1^. Studies about the NCPs of grape berries are less, only a few studies on peptides encoded by the primary miRNA sequences have been reported^6^. All the studies have proven that the NCPs played important functions and cannot be ignored.

In this study, we collected berries of three development stages from ‘Kyoho’ based on EL system^15^. Then the total protein was extracted and filtered through the 10 Kda ultrafiltration tube. After peptides desalination, the mass spectrometry and chromatography experiments were performed. In order to identify more NCPs, we adopt a combination of standard database and customized database. The standard database was retrieved from Ensembl protein database from grape and *Arabidopsis thaliana.* The customized database was constructed based on six-frame translation. The peaks studio software was used to scan the mass spectrometry to the two databases mentioned above. In total, we identified 188 and 2,021 non redundant peptides from the *Arabidopsis thaliana* and *Vitis vinifera* L. protein database in Ensembl/URGI and individualized peptidogenomic database, respectively. Then the chromosome distribution of NCPs and CPs，as well as the origins of NCPs were analyzed. The results indicated that the NCPs were widespread in the grape genome, and the distribution pattern of NCPs and CPs on chromosome were different. In order to analyze the function of NCPs, we also compared the location of NCPs with QTLs, LTR retrotransposons and domestication selection. The results showed that about 94% NCPs were in the QTLs, such as development, intrinsic quality, disease resistant and fruit quality. Finally, we also analyzed whether these NCPs were developmental specificity. These large-scale identified NCPs provide important information for our understanding of these small molecules in grapes.

**Results**

**Peptidogenomic operation process for NCP identification**

To detect the NCPs in grape berry, we adopted a peptidogenomic operation process according to Fig. 1a. The total protein of grape berry was extracted according to experimental method. Then a 10 Kda cutoff filters was used to enrich the peptides from the total protein of grape berry and the C18 cartridges was used to desalt peptide mixtures. In order to obtain the endogenous peptides widely present in grapes, the peaks studio was used to search the produced mass spectrum dataset against the *Arabidopsis thaliana* and *Vitis vinifera* L. protein database in Ensembl/URGI and an individualized peptidogenomic database. The construction of individualized peptidogenomic database was based on the six-frame translation of grape genome sequences (Fig. 1b). Accordingly, a 4.07 x 10^9^ (B) individualized peptidogenomic database (45,664,424 sequences) was obtained.


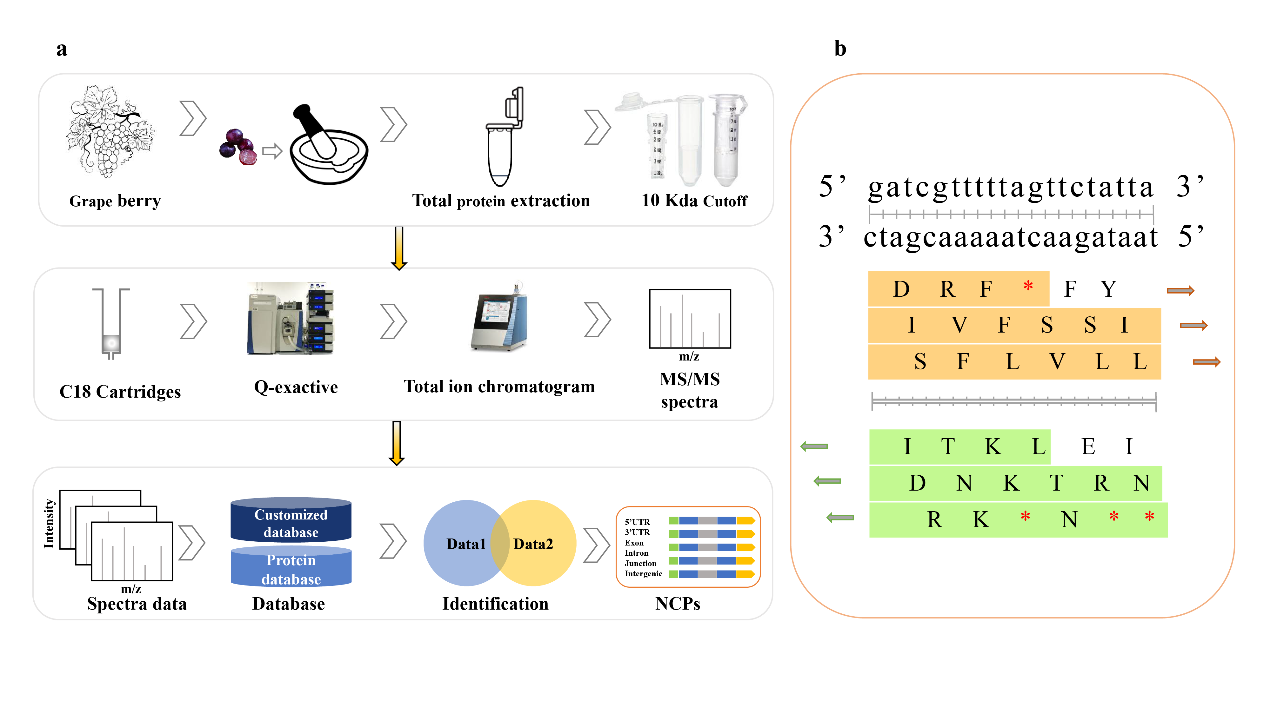


**Fig. 1.** The workflow chart of grape NCPs identification

**a:** The workflow of grape NCPs identification. The endogenous peptides were extracted through conventional methods. Then the peptides were enriched from the total protein of grape berry through a 10 Kda cutoff filters and the C18 cartridges was used to desalt peptide mixtures. The polypeptides were separated by capillary high performance liquid chromatography and analyzed by MASS spectrometry with Q-Exactive Plus Mass spectrometer. The peaks studio was used to search against the URGI protein database for grape and Ensembl protein database for Arabidopsis, and the individualized peptides database to identify peptides. **b:** Construction of grape customized peptidogemic database. The sixpack of emboss-6.6.0^16^ was used to construct potential peptide database. The peptide terminated at the stop codon and the next peptide was then started after the previous stop codon. The location information of putative peptide was recorded and stored in FASTA format.

**Identification and distribution mod of CPs and NCPs in grape**

In sum, we identified 188 and 2,021 non-repetitive peptides from the grape-*Arabidopsis* Ensembl/URGI protein database and individualized peptidogenomic database, respectively (Table S1 and S2). Of these, 1,897 NCPs and 183 CPs were identified assigning to a single genomic locus (Fig. 2a; Table S3 and S4). The median length of both CPs and NCPs was 10.1 and 10.6, and there was no significant difference in length of CPs and NCPs (Fig. 2b). About 90% of CPs and NCPs contained less than 17 and 20 amino acids, respectively. Moreover, the average molecular weight (AMV) of NCPs was 1,220.87 Da, of which peptides with molecular weight less than 2,500 Da account for 95.20% (1925). While, the AMV of CPs was 1,163.376 Da, of which 96.80% (182) of the peptides have a molecular weight less than 2500 (Fig. 2c and 2d). These results indicated that NCPs is an important component of plant proteins.


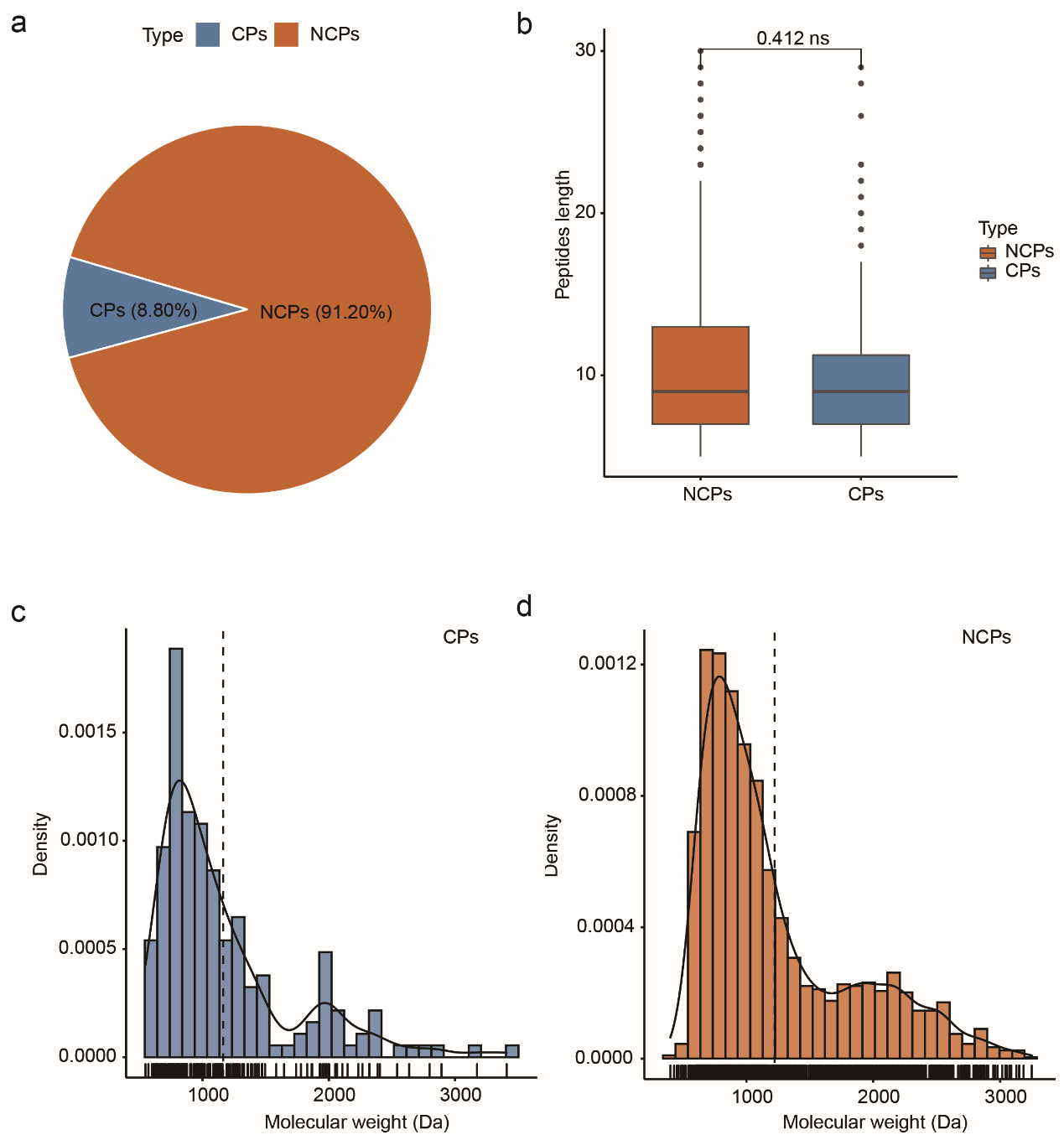


**Fig. 2.** Overview of peptides identification in grape

**a:** Pie chart of CPs and NCPs number identified. **b:** Boxplot of length distribution of CPs and NCPs. Hypothesis testing with Wilcox test (**p* < 0.05). **c:** The statistics of CPs molecular weight (188). **d:** The statistics of NCPs molecular weight (2,021).

The distribution of both NCPs and CPs on grape chromosomes was uneven (Fig. 3a). Moreover, 124 intensive regions (defined by 1Mb windows) were identified (Fig. 3a). Among which, 22 CPs of intensive regions containing 28 peptides (18.18%) were obtained, while 102 NCPs of intensive regions containing 721 peptides (37.61%) were found (Fig. 3a). Among these intensive regions, four regions located in chr1, chr16, chr18 and chr19 were shared by both CPs and NCPs. Meanwhile, there was no correlation between the number of CPs and chromosome length (R=0.21, *p*=0.39), while the number of NCPs and chromosome length were correlated (R=0.88, *p*=7.4e-07) (Fig. 3b).

To estimate the coverage of peptides over the genome, the interval between the adjacent peptides (Kb) were calculated. The results showed that 87.67% (1,742) NCPs were within 500 Kb of each other, while only 14.89% (21) CPs were within 500 Kb of each other (Fig. 3c). Moreover, 32.14% (645) NCPs were founded located within 2 Kb to the adjacent canonical translation start site (TSS) (Fig. 3d). These results indicated that the NCPs were widespread in the grape genome, and the distribution pattern of NCPs and CPs on chromosome were different.

Previous studies have showed that most endogenous peptides start with non-AUG codon^1,17^. In this study, the mRNA sequence of NCPs and CPs revealed that dominant NCPs and CPs were non-AUG TSSs (Table S3 and Table S4). Although it is well known that the translation initiation site in eukaryotes is AUG, our results revealed that non-AUG initiated translation is also widespread. The results were consisted with mass spectrometric analysis^18^.


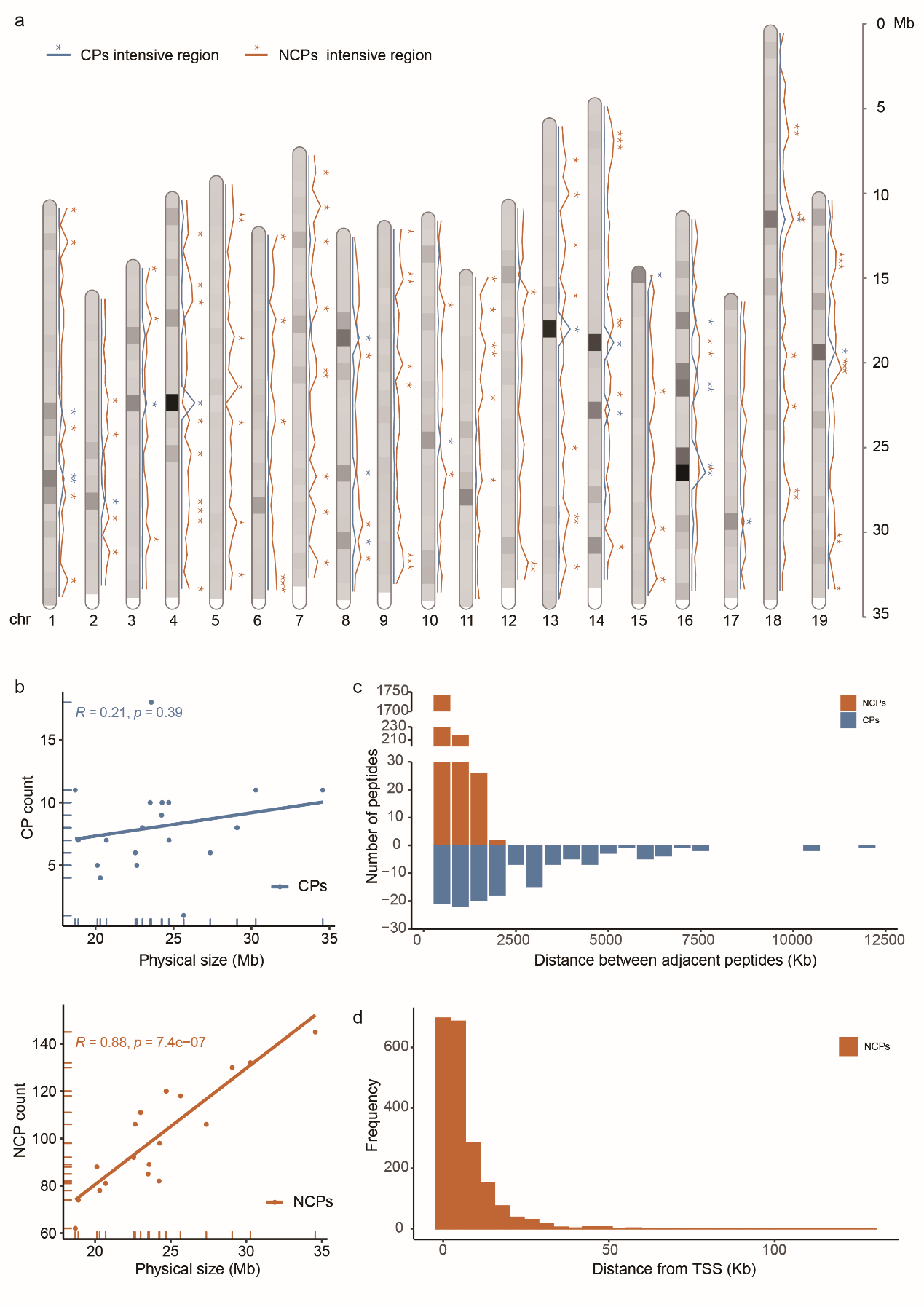


**Fig. 3.** Distribution of CPs and NCPs in grapes

**a:** The distribution statistics of grape genome CPs and NCPs. The blue line represents CPs and the orange line represents NCPs. The * represents hotspot regions (window size = 1 Mb). **b:** The correlations between CPs and NCPs count and physical size with Levenberg-Marquardt (LM) method. **c:** Distance distribution of two neighboring CPs or NCPs. **d:** Histogram of the distances from each NCPs and CPs to the neighboring TSS.

**Analysis of NCPs originated from the coding region or the non-coding region**

The origins analysis of NCPs showed that 49.5% (994) were lactated on the reverse strand in grape (Fig. 4a). Meanwhile, the gene resource analysis showed that 1953 (97.31%) NCPs were originated from intergenic region, 24 (1.20%) were originated from intronic region, 13 (0.65%) were originated from upstream region (The upstream is defined as 1-kb away from transcription start site), 9 (0.45%) NCPs were originated from downstream region, 5 (0.25%) were originated from exonic region, 2 (0.10%) were originated from 5’UTR region and 1 (0.050%) were originated from 3’UTR region (Fig. 4b). These results suggest that the non-coding sequences were translated. Length analysis indicated that NCPs derived from exonic region were longer than that derived from 3’UTR region (Fig. 4c). The molecular weight analysis results showed that 70% NCPs were less than that 1,300 Da, and the AMV of NCPs originated from Upstream, 5'UTR, exonic, intergenic, intronic, 3'UTR and downstream region were insignificant difference (Fig. 4d and Table S5). The mass number/charge number (m/z) of NCPs derived from upstream and intergenic region, upstream and downstream region, intronic and downstream region were significant different (Fig. 4e).

To verify the NCPs identified in the study, the NCPs were compared with published long non-coding RNA (lncRNAs) sequencing data from grape^19^. The results showed that 22 NCPs were derived from the lncRNAs (Table S6).


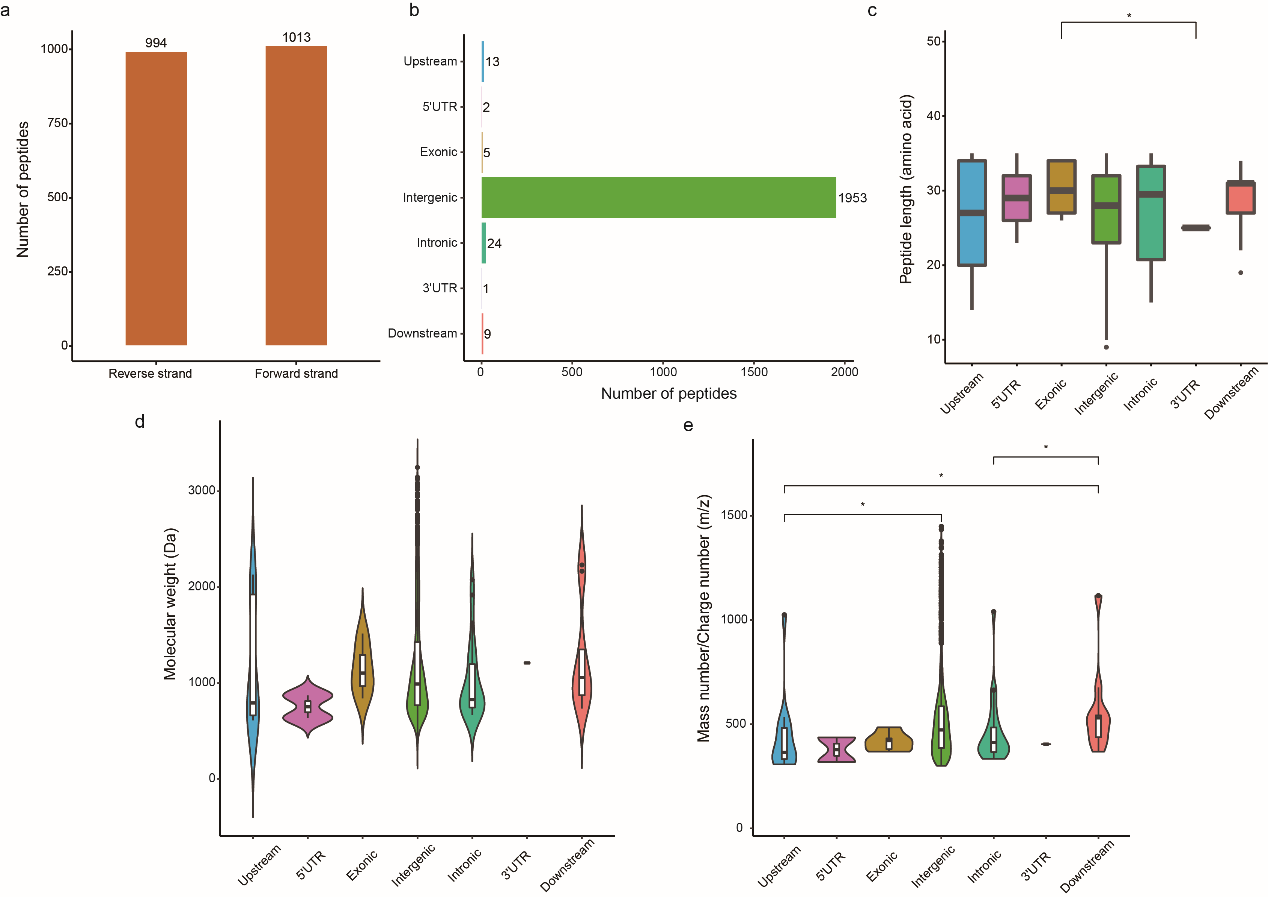


**Fig. 4.** Features of grape NCPs

**a:** Histogram of the number of sense and antisense strands NCPs. **b:** Number statistics of NCPs originated from different regions. **c-e:** Length, molecular wight and mass number/charge number of NCPs acquired from different regions. Hypothesis testing with Wilcox test (**p* < 0.05).

**Verification of NCPs**

To determine whether non-conventional regions harbored NCPs have been transcribed, we have investigated the overlap of NCPs locations with the PacBio SMRT Iso-seq data from ‘Kyoho’ grape berries which deposited in SRA (PRJNA790655) (https://dataview.ncbi.nlm.nih.gov/object/PRJNA790655), and the results showed that 87 NCPs were overlapped with the RNA-seq reads (Table S7). Then 10 NCPs were randomly selected for display with IGV^20^ (Fig. 5). These results indicated that the NCPs were indeed transcribed.


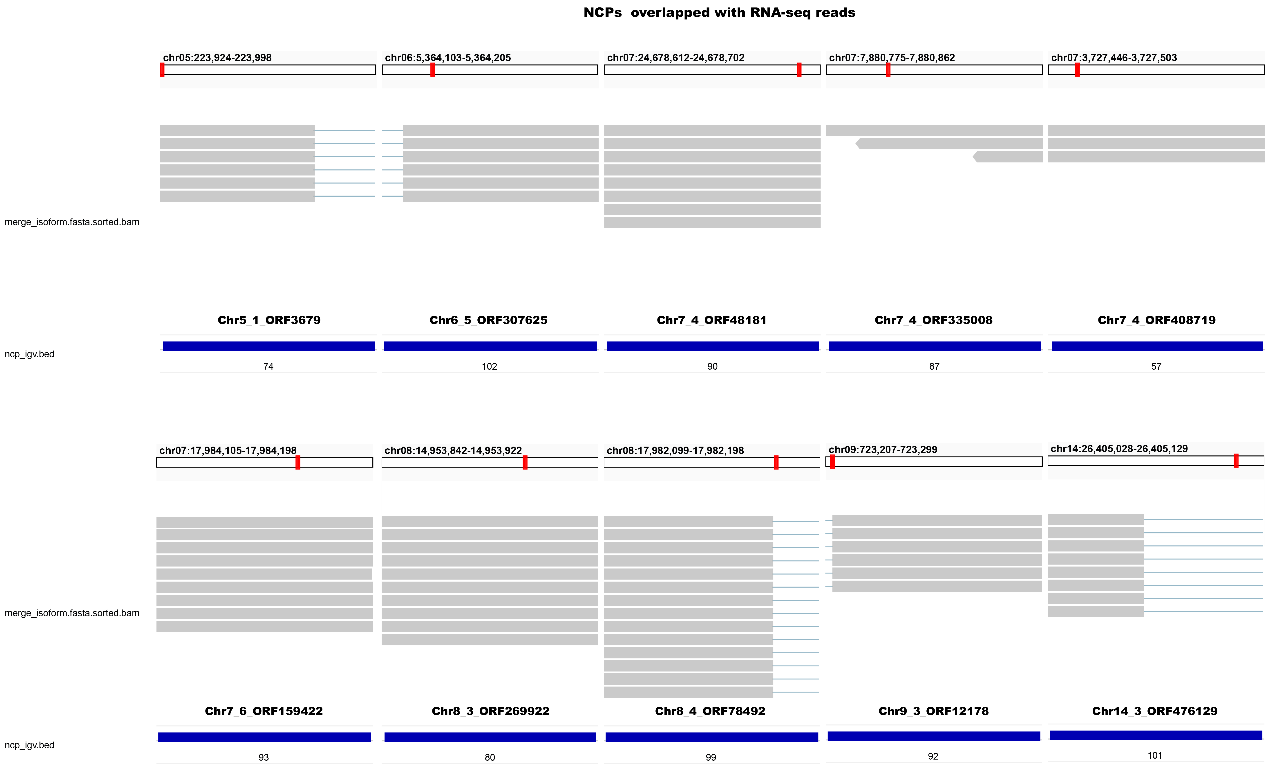


**Fig. 5. The overlap of NCPs and RNA-seq data reads**

The overlap of NCPs locations with the PacBio SMRT Iso-seq data from ‘Kyoho’ grape berries. The red box represents the position of NCPS on the chromosome.

**NCPs located in regions associated with QTLs**

In grape, there are many quantitative trait loci (QTL) associated various traits have been identified^21^, such as QTLs related to fruit quality, stress, disease-resistant, intrinsic quality and leaf physiological index (Table S8). Then the relationship between QTLs and NCPs were investigated. The results showed that most NCPs (94%) were located in the QTLs (Fig. 6a; Table S9). Among QTLs related to development, most NCPs were in regions related to ‘flowering’ and ‘growth’. In the intrinsic quality category, the top two terms were ‘tannins’ and ‘anthocynins’. In the disease resistant category, ‘Chlorosis’ and ‘downy mildew’ were the dominant terms. In the fruit quality category, ‘berry weight’ and ‘seed fresh weight’ were the main terms. There were also many NCPs located in region with ‘water use efficiency’, ‘drought stress’ and ‘leaf area’. The results showed that these NCPs may play a potential role in the regulation of plant growth, development and resistance.

Long terminal repeat retrotransposons (LTR-RTs) are ubiquitous and dominant repeat elements in plants genomes and play a crucial role in genomic diversity, phenotypic variations and evolution^22^. All the LTR-RTs were identified through LTR_retriever (Table S10). Then the relationship between NCPs and LTR-RTs was investigated, the results showed that a total of 47 NCPs were identified located in LTR-RTs regions (Table S11). While the function of these NCPs still needs further explored.


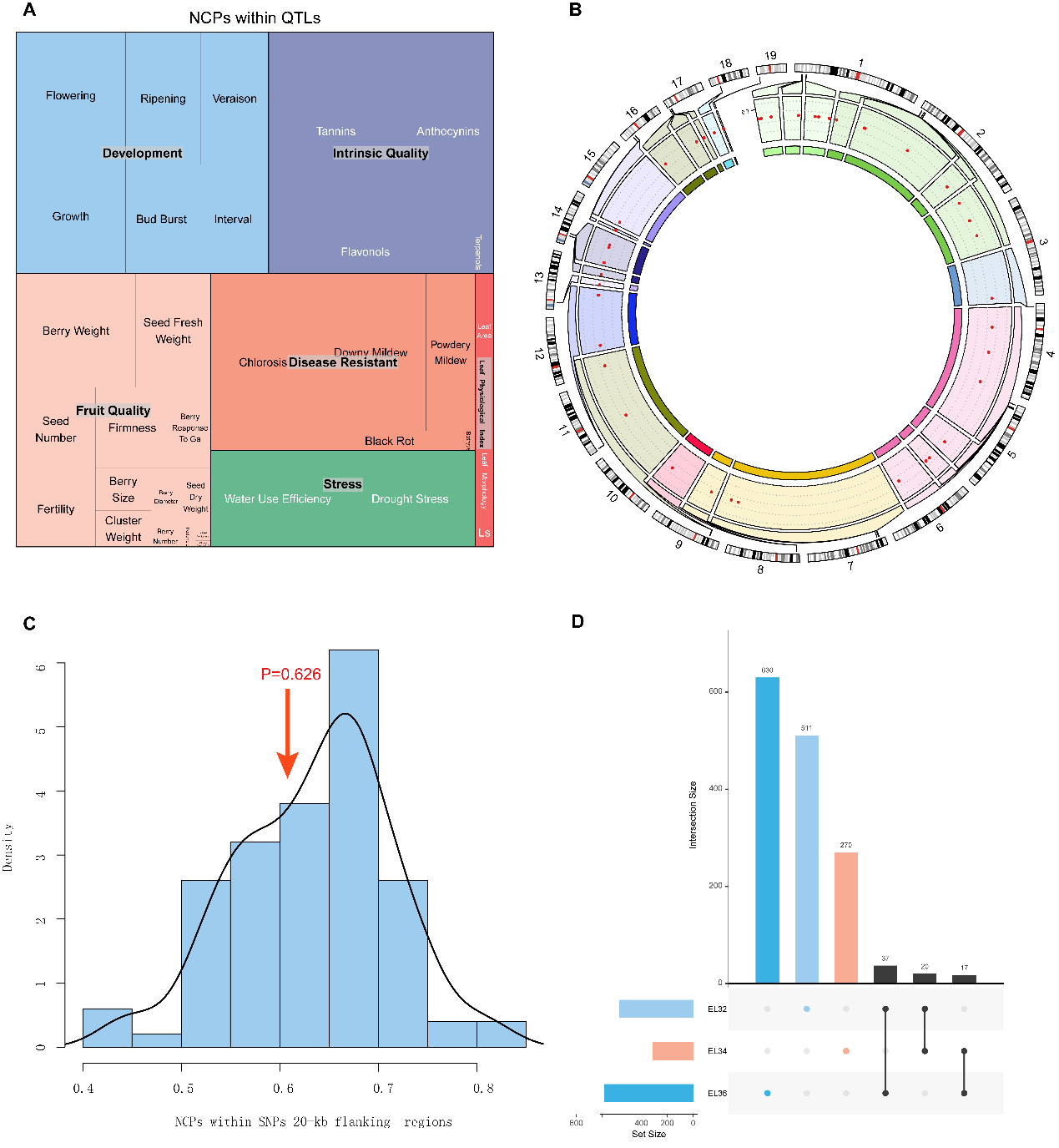


**Fig. 6.** Quantitative Trait Loci and selective regions associated with grape NCPs and comparison of development periods

**a:** NCPs associated with QTLs. **b:** NCPs overlapped with selective regions. The red dots indicate the zoom sectors of NCPs located in the selective regions. **c:** NCPs within SNPs 20-kb flanking regions. The ‘pnorm’ function in R was used to calculate the upper-tail test *p* values (lower.tail = FALSE). **d:** Comparison of NCPs in different development periods.

**The distribution of NCPs in selective regions and phenotypic variation regions**

Grapes, one of the earliest domesticated fruit crops, are widely cultivated for both fresh consumption and winemaking. During the domestication of grape, wild species are genetically selected for traits that are beneficial to humans as a source of food or material, including flower sex, berry size, sugar content, and berry color. Here, the relationship between NCPs and domestication such as selective sweeps for domesticated table and wine grape, wild and domesticated grapes, berry edibility and stress resistance were investigated^23-25^. The results showed that 42 NCPs were screened to be overlapped with the selective regions (Fig. 6c, Table S12). However, how NCPs affect domestication traits, and what is the mechanism of action, needs further verification.

Because of the existence of linked inheritance, the relationship between the 10-kb interval upstream and downstream of SNPs related to the investigated traits and NCPs was also investigated^23-24,26^. In total, 61 NCPs were identified overlapped with the 10-kb interval upstream and downstream of SNPs related to the investigated traits which include berry shape, berry weight, berry color, flower sex and intrinsic quality, etc (Table S13). Compared to randomly generated genome fragments of the same length and the same number on the same chromosome (see Methods), the plant traits-associated SNPs was not significant enrichment in the NCPs regions (*p* < 0.626, upper-tail test; Fig. 6c). These results were not similar with the pattern of NCPs in maize^1^, which maybe indicated that the NCPs in different species have their unique characteristics.

**NCPs are developmental specificity in ‘Kyoho’ grape**

To explore whether the NCPs were developmental specificity, all the NCPs deprived from EL32, EL34, EL36 which represent ‘beginning of bunch closure’, ‘berries begin to soften’ and ‘berries with intermediate brix values’, respectively, were compare^15^. The results showed that there were 511, 270, and 630 NCPs derived from EL32, EL34 and EL36, respectively (Fig. 6d). Among the NCPs there were 20 were shared by EL32 and EL34, 37 were shared by EL32 and EL36, 17 were shared by EL34 and EL36 (Table S14). Herein, we can draw a conclusion that the NCPs were developmental specificity.

**DISCUSSION**

Mass spectrometry (MS) is an efficient technique for peptides detection in proteomics research. The standard operating procedure is to map the mass spectrometry data of the MS experiment to the annotated genes. MS differ from other experiments such as ribosome profiling, can directly verify the translation of the transcripts^14^. Interestingly, so far, there is still a large number of MS fragmentation spectra that have not been identified and mainly because that some of the spectra are belong to the unannotated peptides in proteomics studies. Peptidogenomics is a feasible method to identify the unannotated peptides which combines the proteomics with six-frame translation of a genome^27-28^. An individualized database constructed by six-frame translation in a genome-wide scale is able to identify all possible peptides effectively^29-30^. Although peptidogenomics has been successfully applied in identifying peptides in micro-organisms and humans, it has rarely been applied in plants besides maize^1,12,31-33^. In the study, the peptidomics and the individualized peptidogenomic database constructed by six-frame translation of the URGI/ENsembl protein databases of grape and *Arabidopsis* were combined. As far as we know, this is the first published work on the peptidogenomic analysis of NCPs in grape. 1,897 NCPs and 183 CPs were identified in the customized database and URGI/ENsembl protein databases. The study demonstrated that the integrative of MS and peptidogenomic was an effective method to detect CPs and NCPs. In our results most peptides were NCPs, indicating that those previously thought untranslated sequences, such as 5’UTR, intergenic, intronic and 3’UTR are capable of translation.

Recently, more and more studies have proven that the proteins encoded by lncRNAs play an import role in cellular processes. Such as HOXB-AS3 a putative lncRNA encoded peptide which could suppress colon cancer growth^34^. A peptide encoded by lncRNA could enhance SERCA activity in muscle through activating the calcium pump^35^. The newly putative lncRNA HBVPTPAP encoded peptide was reported to induce the apoptosis of hepatocellular carcinoma cells by regulating JAK/STAT signaling pathways^36^. In addition, four lncRNA-encoded peptides identified in moss were proven involved in regulation of growth and differentiation by overexpression and inhibitory expression experiments^37^. In our study, 22 NCPs were found to be derived from lncRNA in grape (Table S6). Although the function of these NCPs was unclear, it lay a foundation for further study of these small peptides encoded by lncRNA.

The upstream ORFs peptides have attracted attention because of their function to control the translation of downstream ORF^38-40^. For example, an upstream open reading frame was reported to repress the expression of a tomato NAC domain transcription in a peptide sequence-dependent manner^41^. Upstream open reading frame mediated translation of WNK8 is essential for *Arabidopsis* response to ABA^42^. In this study, 13 NCPs were identified derived from upstream in grapes, and 2 NCPs were identified derived from 5’UTR. Compared to 5’UTR, peptides derived from the 3’UTR region have traditionally been overlooked. Until recently, the peptides derived from 3’UTR region were identified in moss^37^. In the study, we only identified one NCPs derived from 3’UTR, but their biological function shouldn’t be ignored.

Although many QTLs have been identified in grape, few studies pay attention to the relationship between QTLs and NCPs. In maize, studies have proved that the QTLs were highly associated with NCPs. Clark *et al*. (2006)^43^ showed that the intergenic sequences of maize played a pleiotropic effect on morphology. Castelletti *et al*. (2014)^44^ found that the NCPs which located in *Vgt1* QTL of maize flowering time was associated with the methylation state. Wang *et al*. (2020)^1^ also reported that the NCPs were significantly enriched within plant traits-associated QTL regions. Therefore, associating NCPs with QTLs is an effective method to study their function. In our study, we found that most NCPs (94%) were located in the QTLs, such as development, intrinsic quality, fruit quality, disease resistant, stress and leaf physiological. These results indicating that the NCPs play an important function in regulating these traits. LTR-RTs are believed crucial to maintain chromatin structure and centromeric function, as well as gene expression regulation in the host genome^45^. In the study, the LTR-RTs were identified and the relation between NCPs and LTR-RTs were analyzed. The function of the NCPs located in the LTR-RTs region need further exploration. Domestication is widespread in cultivated crops, herein, screening for domestication-related genes is very helpful for our understanding of evolution, which will also advance the domestication of crops. In the study 42 NCPs were found overlapped with the selective regions, indicating the underlying functional sites for the evolution of grape.

By comparing the overlap of NCPs at different developmental stages, no peptides were continuously expressed throughout the development period of grape berries. These results indicated that the NCPs may play instant and special function in different biological process. These were also in consist with that most peptides were function as signaling molecules in biological processes. For example, the cystein-rich peptides LAT52, LeSTIG1 and MAPK participated in self-incompatibility responses^2^.

Taken together, this is the first study using a peptidogenomic approach to identify NCPs on a large scale in the grape genome. The results indicates that mounts of previously considered untranslated regions such as intergenic, intronic, upstream ORFs, 5’UTR, 3’UTR and downstream ORFs are translated and have potential function in the biological process. These funding also provides some reference for discovering some new functional genes in grape.

**Materials and Methods**

**Plant Material**

Three ‘Kyooho’ grape threes with the same vigor were selected in 2021 spring at the farm of Henan University of Science & Technology, Luoyang, China. The grape berries at EL32, EL34 and EL36 according EL system^15^ were harvested and flash-frozen with liquid nitrogen, then stored them at -80℃.

**The extraction of peptide**

Grape berries samples were weighed (3.0 g) and grounded to powder in low-temperature. Then the tissue powder was transferred to an EP tube with 8M Urea lysate, and sonicated for 6min (Ultrasonic power 20%, 2s ON/3s OFF) on ice to break the tissue. Tubes were centrifuged for 15 minutes (at 12,000 rpm,4 ℃), then transferred the supernatant to a 10 KDa ultrafiltration tube for ultrafiltration separation, and centrifuged at 1,1000 rpm at 4°C.

**Peptide desalination**

Firstly, a C18 membrane-packed column were prepared and centrifuged three times with 40 μL methanol to active it. Secondly, the column was balanced with 40 μL Nano-HPLC Buffer A for three times. The dried polypeptide extracts were re-dissolved in Nano-HPLC Buffer A and centrifuged three times on the balanced C18 column. Then, the C18 column were centrifuged three times with 40 μL Nano-HPLC Buffer A for desalting. Finally, the C18 column was centrifuged twice with 40 μL elution phase Buffer B to collect the desalinated polypeptides.

**LC-MS/MS analysis**

The dried polypeptide extracts were re-dissolved in Nano-HPLC Buffer A and separated by Nano-HPLC liquid system UltiMate 3000 RSLCnano (Thermo Fisher Scientific, MA, USA). Among, solution A is 0.1% formic acid-water solution, and solution B is 0.1% formic acid-acetonitrile solution. The Trap column was balanced with 100 % solution A at 3 μL/min (RP-C18, Agilent). Then the samples were loaded by an automatic sampler and combined with the trap column, and separated on an analysis column at a flow rate of 300 nL/min on a 75 μm × 150 mm (RP-C18, New Objective, USA) column. Peptides were separated by capillary high-performance liquid chromatography, while MASS spectrometry was performed with a Q-Exactive Plus mass spectrometer (Thermo Fisher Scientific). The detection methods were as follows: after calibration with the standard calibration solution, the mother solution was scanned through the data dependent acquisition (DDA) mode (350-2000 m/z). Then the 20 strongest fragment profiles (MS2 scan) were collected after high energy collision dissociation (NCE energy 28, dynamic exclusion time: 25 s). We set the resolution of MS1 to 70,000 at M/Z 200, and the AGC target to 3e6, the maximum injection time to 100 ms. Meanwhile, we also set the resolution of MS2 to 17,500, the AGC target to 1e5, and the maximum injection time to 50 ms.

**Construction of peptide database**

The genome of grape^46^ and *Arabidopsis*^47^ was downloaded from URGI (https://urgi.versailles.inra.fr/files/Vini/Vitis%2012X.2%20annotations/12Xv2_grapevine_genome_assembly.fa.zip) and Ensembl (http://ftp.ensemblgenomes.org/pub/ plants/release-51/fasta/arabidopsis_thaliana/dna/). The sixpack of emboss-6.6.0^16^ was used to construct potential peptide database. The peptide terminated at the stop codon and the next peptide was then started after the previous stop codon The location information of putative peptide was recorded and stored in FASTA format.

**Peptides identification by peaks studio**

The peaks studio-5.3 (Bioinformatics Solutions, Inc.) was used to search against the URGI protein database for grape^46^ (https://urgi.versailles.inra.fr/files/Vini/Vitis%2012X.2%20annotations/vitviv2.pep.fasta.zip) and Ensembl protein database for *Arabidopsis*^47^ (http://ftp.ensemblgenomes.org/ pub/plants/release-51/fasta/arabidopsis_thaliana/pep/), and the individualized peptides database to recognize peptides. The type of peptide-producing sequence was determined with bedtools-v2.25.0^48^. Peptides derived from annotated CDs were defined as CPs. The intergenic regions, UTRs regions, reading frames different from annotated CDs, intronic regions peptides were defined as NCPs.

**Peptides distribution on chromosome**

Peptide density on chromosome was calculated by R package RIdeogram::genomicDensity^49^ with a sliding window size of 1e6. The distance between peptides to the adjacent TSSs was calculated by Python script (https://github.com/Peims/Calculate-the-distance-between-peptide-and-adjacent-stop-codon) based on the grape genome annotation^46^ (https://urgi.versailles.inra.fr/files/Vini/Vitis%2012X.2%20annotations/Vitis_vinifera_gene_annotation_on_V2_20.gff3.zip). Then the distance between the peptides and adjacent to TSSs was used to draw the frequency plot.

**Verification of NCPs**

The PacBio SMRT Iso-seq data from ‘Kyoho’ grape berries which deposited in SRA (PRJNA790655) (<https://dataview.ncbi.nlm.nih.gov/object/PRJNA790655>) were downloaded, then the bedtools-v2.25.0^48^ was used to calculate the overlap between NCPs and RNA-seq reads. The NCPs overlapped with RNA-seq reads were demonstrated using IGV-2.11.9^20^.

**Association of NCPs with QTLs, LTR retrotransposons and domestication selection**

The QTLs related to 34 traits including development, intrinsic quality, fruit quality, disease resistant, stress and leaf physiological index were collected (Table S8)^21^. The LTR retrotransposons were detected and retrieved by LTRharverst^50^ and LTR_finder^51^ from grape genome^46^ (https://urgi.versailles.inra.fr/files/Vini/Vitis%2012X.2%20annotations/12Xv2_grapevine_genome_assembly.fa.zip) (Table S10). The domestication intervals of grape fruits were collected from both wild and domesticated grapevines^23-25^. The NCPs which were intersect with the QTLs, LTR retrotransposons and domestication intervals were selected as candidate NCPs.

**Association analysis of NCPs with SNPs**

SNPs were collected from both wild European and domesticated grapevines^23-24,26^. 100 genomic sequences were randomly generated as background, each random sequence had the same characteristics as NCPs, including total quantity, distribution modes on different chromosomes, and peptide length distribution^1^ (Fig. S1). The mean and SD of the normal distribution of NCPs were calculated using the100 random genomic sequences within SNPs 20-kb flanking regions. The ‘pnorm’ function in R was used to calculate the upper-tail test p values (lower.tail = FALSE); the p-value represented the probability that the observed value exceeds the expected distribution.

**Availability of supporting data**

LC-MS/MS data from ‘Kyoho’ grape berry EL32, EL34 and EL36 were deposited in the iProX (integrated proteome resources) (<https://www.iprox.org/>) database under accession number IPX0003909000 (https://www.iprox.cn/page/project.html?id=IPX0003909000). The release time is 2022-12-29 00:00:00. Data sets during the current study and/or analysis may be provided by the corresponding author upon reasonable request

**Acknowledgments**

This work was financially supported by National Key Research and Development Program of China (2018YFD1000105), Natural Science Foundation of China (NSFC: U1904113) and Program for Innovative Research Team (in Science and Technology) in University of Henan Province (21IRTSTHN021), Program for Science & Technology Innovation Talents in Universities of Henan Province (21HASTIT035).

**Conflict of interest statement**

The authors declare that the research was conducted in the absence of any commercial or financial relationships that could be construed as a potential conflict of interest.

**Contributions**

D-L.G. conceived the original screening and research plans; M-S.P. and H-N.L. performed the experiments using LC-MS/MS methods and bioinformatic analysis; M-S.P. wrote the manuscript; Y-H.Y. and T.-L. W gave advices for this study and helped to revise the manuscript. All authors read and approved the final manuscript.

**Figure legends**

**Fig. 1.** The flow chart of grape NCPs identification

**a:** The workflow of grape NCPs identification. The endogenous peptides were extracted through conventional methods. Then the peptides were enriched from the total protein of grape berry through a 10 Kda cutoff filters and the C18 cartridges was used to desalt peptide mixtures. The polypeptides were separated by capillary high performance liquid chromatography and analyzed by MASS spectrometry with Q-Exactive Plus Mass spectrometer. The peaks studio was used to search against the URGI protein database for grape and Ensembl protein database for Arabidopsis, and the individualized peptides database to identify peptides. **b:** Construction of grape customized peptidogemic database. The sixpack of emboss-6.6.0^16^ was used to construct potential peptide database. The peptide terminated at the stop codon and the next peptide was then started after the previous stop codon. The location information of putative peptide was recorded and stored in FASTA format.

**Fig. 2.** Overview of peptides identification in grape

**a:** Pie chart of CPs and NCPs number identified. **b:** Boxplot of length distribution of CPs and NCPs. Hypothesis testing with Wilcox test (**p* < 0.05). **c:** The statistics of CPs molecular weight (188). **d:** The statistics of NCPs molecular weight (2,021).

**Fig. 3.** Distribution of CPs and NCPs in grapes

**a:** The distribution statistics of grape genome CPs and NCPs. The blue line represents CPs and the orange line represents NCPs. The * represents hotspot regions (window size = 1 Mb). **b:** The correlations between CPs and NCPs count and physical size with Levenberg-Marquardt (LM) method. **c:** Distance distribution of two neighboring CPs or NCPs. **d:** Histogram of the distances from each NCPs and CPs to the neighboring TSS.

**Fig. 4.** Features of grape NCPs

**a:** Histogram of the number of sense and antisense strands NCPs. **b:** Number statistics of NCPs originated from different regions. **c-e:** Length, molecular wight and mass number/charge number of NCPs acquired from different regions. Hypothesis testing with Wilcox test (**p* < 0.05).

**Fig. 5. The overlap of NCPs and RNA-seq data reads**

The overlap of NCPs locations with the PacBio SMRT Iso-seq data from ‘Kyoho’ grape berries. The red box represents the position of NCPS on the chromosome.

**Fig. 6.** Quantitative Trait Loci and selective regions associated with grape NCPs and comparison of development periods

**a:** NCPs associated with QTLs. **b:** NCPs overlapped with selective regions. The red dots indicate the zoom sectors of NCPs located in the selective regions. **c:** NCPs within SNPs 20-kb flanking regions. The ‘pnorm’ function in R was used to calculate the upper-tail test *p* values (lower.tail = FALSE). **d:** Comparison of NCPs in different development periods

**Supplementary Fig. S1.** Statics analysis for the distribution of within SNPs flanking regions ratio between NCPs and random sequences

**Supplementary Table S1.** Non-repetitive peptides acquired from the customized grape peptidogenomic database

**Supplementary Table S2.** Non-repetitive peptides acquired from the grape and *Arabidopsis* protein database

**Supplementary Table S3.** NCPs identified in Grape

**Supplementary Table S4.** CPs identified in Grape and *Arabidopsis*

**Supplementary Table S5.** The NCPs location and characteristics of grape

**Supplementary Table S6.** The NCPs derived from published grape lncRNAs

**Supplementary Table S7.** The NCPs overlapped with RNA-seq data

**Supplementary Table S8.** QTLs associated with various traits

**Supplementary Table S9.** NCPs derived from QTLs

**Supplementary Table S10.** LTR_RTs identified in grape

**Supplementary Table S11.** NCPs derived from LTR-RTs

**Supplementary Table S12.** NCPs overlapped with selective regions

**Supplementary Table S13.** NCPs overlapped with 10-kb interval upstream and downstream of SNPs related to the investigated traits

**Supplementary Table S14.** NCPs derived from different development stages

**References**

1 Wang, S. et al. Large-scale discovery of non-conventional peptides in Maize and *Arabidopsis* through an integrated peptidogenomic pipeline. *Mol. Plant* **13**, 1078-1093 (2020).

2 Qu, L. J. et al. Peptide signaling during the pollen tube journey and double fertilization. *J. Exp. Bot.* **66**, 5139-5150 (2015).

3 Casson, S. A. et al. The POLARIS gene of Arabidopsis encodes a predicted peptide required for correct root growth and leaf vascular patterning. *Plant Cell* **14**, 1705-1721 (2002).

4 Narita, N. N. et al. Overexpression of a novel small peptide ROTUNDIFOLIA4 decreases cell proliferation and alters leaf shape in *Arabidopsis thaliana*. *Plant J*. **38**, 699-713 (2004).

5 De Coninck, B. et al. Mining the genome of Arabidopsis thaliana as a basis for the identification of novel bioactive peptides involved in oxidative stress tolerance. *J. Exp. Bot*. **64**, 5297-5307 (2013).

6 Chen, Q. J. et al. A miRNA-Encoded Small Peptide, vvi-miPEP171d1, Regulates Adventitious Root Formation. *Plant Physiol*. **183**, 656-670 (2020).

7 Lauressergues, D. et al. Primary transcripts of microRNAs encode regulatory peptides. *Nature* **520**, 90-93 (2015).

8 Vale, M. et al. Exogenous application of non-mature miRNA-Encoded miPEP164c inhibits proanthocyanidin synthesis and stimulates anthocyanin accumulation in grape berry cells. *Front. Plant Sci*. **12**, 706679 (2021).

9 Mackowiak, S. D. et al. Extensive identification and analysis of conserved small ORFs in animals. *Genome Biol*. **16**, 179 (2015).

10 Aspden, J. L. et al. Extensive translation of small Open Reading Frames revealed by Poly-Ribo-Seq. *Elife* **3**, e03528 (2014).

11 Galindo, M. I. et al. Peptides encoded by short ORFs control development and define a new eukaryotic gene family. *PLoS Biol.* **5**, e106 (2007).

12 Liang, Y. et al. Genome-Wide Identification and Characterization of Small Peptides in Maize. *Front Plant Sci*. **12**, 695439 (2021).

13 Lease, K. A. & Walker, J. C. The *Arabidopsis* unannotated secreted peptide database, a resource for plant peptidomics. *Plant Physiol*. **142**, 831-838 (2006).

14 Makarewich, C. A. & Olson, E. N. Mining for Micropeptides. *Trends Cell Biol*. **27**, 685-696 (2017).

15 Coombe, B. G. Growth Stages of the Grapevine: Adoption of a system for identifying grapevine growth stages. *Aust. J. Grape Wine Res.* **1**, 104-110 (1995).

16 Rice, P., et al. EMBOSS: the European Molecular Biology Open Software Suite. *Trends Genet*. **16**, 276-277 (2000).

17 Starck, S. R. et al. Translation from the 5’ untranslated region shapes the integrated stress response. *Science* **351**, aad3867 (2016).

18 Na, C. H. et al. Discovery of noncanonical translation initiation sites through mass spectrometric analysis of protein N termini. *Genome Res.* **28**, 25-36 (2018).

19 Zhang, H. L. et al. Genome-wide identification and characterization of long non-coding RNAs involved in grape berry ripening. *J. Berry Res.* **10**, 475-496 (2020).

20 Thorvaldsdóttir, H., Robinson, J.T., & Mesirov, J.P. Integrative Genomics Viewer (IGV): high-performance genomics data visualization and exploration. *Brief Bioinform*. **14**, 178-192 (2013).

21 Delfino, P. et al. Selection of candidate genes controlling veraison time in grapevine through integration of meta-QTL and transcriptomic data. *BMC Genom*. **20**, 739 (2020).

22 Ma, T. et al. Development of molecular markers based on LTR retrotransposon in the Cleistogenes songorica genome. *J. Appl. Genet*. (2021).

23 Liang, Z., et al. Author Correction: Whole-genome resequencing of 472 Vitis accessions for grapevine diversity and demographic history analyses. *Nat. Commun.* **11**, 2341 (2020).

24 Kui, L., et al. Identification of Selective Sweeps in the Domesticated Table and Wine Grape (*Vitis vinifera L*.). *Front Plant Sci*. **11**, 572 (2020).

25 Zou, C., et al. Multiple independent recombinations led to hermaphroditism in grapevine. *Proc Natl Acad Sci USA*. 118(15), e2023548118 (2021).

26 Guo, D.L., et al. Genome-wide association study of berry-related traits in grape (*Vitis vinifera L*.) based on genotyping-by-sequencing markers. *Hortic Res*. **6**, 11 (2019).

27 Castellana, N. & Bafna, V. Proteogenomics to discover the full coding content of genomes: a computational perspective. *J. Proteomics* **73**, 2124-2135 (2010).

28 Woo, S. et al. Proteogenomic database construction driven from large scale RNA-seq data. *J. Proteome Res.* **13**, 21-28 (2014).

29 Yang, M. K. et al. Genome annotation of a model diatom *Phaeodactylum tricornutum* using an integrated proteogenomic pipeline. *Mol. Plant.* **11**, 1292-1307 (2018).

30 Nesvizhskii, A. I. Proteogenomics: concepts, applications and computational strategies. *Nat. Methods* **11**, 1114-1125 (2014).

31 Kersten, R.D. et al. A mass spectrometry-guided genome mining approach for natural product peptidogenomics. *Nat. Chem. Biol.* **7**, 794-802 (2011).

32 Mohimani, H. & Pevzner, P.A. Dereplication, sequencing and identification of peptidic natural products: from genome mining to peptidogenomics to spectral networks. *Nat. Prod. Rep.* **33**, 73-86 (2016).

33 Slavoff, S. A. et al. Peptidomic discovery of short open reading frame-encoded peptides in human cells. *Nat. Chem. Biol*. **9**, 59-64 (2013).

34 Huang, J. Z. et al. A Peptide Encoded by a Putative lncRNA HOXB-AS3 Suppresses Colon Cancer Growth. *Mol. Cell* **68**, 171-184 (2017).

35 Nelson, B. R. et al. A peptide encoded by a transcript annotated as long noncoding RNA enhances SERCA activity in muscle. *Science* **351**, 271-275 (2016).

36 Lun, Y.Z. et al. The peptide encoded by a novel putative lncRNA HBVPTPAP inducing the apoptosis of hepatocellular carcinoma cells by modulating JAK/STAT signaling pathways. *Virus Res.* **287**, 198104 (2020).

37 Fesenko, I. et al. Distinct types of short open reading frames are translated in plant cells. *Genome Res.* **29**, 1464-1477 (2019).

38 Jorgensen, R. A. & Dorantes-Acosta, A. E. Conserved Peptide Upstream Open Reading Frames are Associated with Regulatory Genes in Angiosperms. *Frontiers in Plant Science* **3**, 191 (2012).

39 von Arnim, A. G. Jia, Q. & Vaughn, J. N. Regulation of plant translation by upstream open reading frames. *Plant Sci*. **214**, 1-12 (2014).

40 Hsu, P. Y. & Benfey, P. N. Small but mighty: functional peptides encoded by small ORFs in plants. *Proteomics* **18**, e1700038 (2018).

41 Noh, A. L. et al. An upstream open reading frame represses expression of a tomato homologue of *Arabidopsis* ANAC096, a NAC domain transcription factor gene, in a peptide sequence dependent manner. *Plant Biotechnol.* **32**, 157-163 (2015).

42 Li, Z. et al. Upstream Open Reading Frame Mediated Translation of WNK8 Is Required for ABA Response in *Arabidopsis*. *Int. J. mol. Sci.* **22**, 10683 (2021).

43 Clark, R. M. et al. A distant upstream enhancer at the maize domestication gene tb1 has pleiotropic effects on plant and inflorescent architecture. *Nat. Genet.* **38**, 594-597 (2006).

44 Castelletti, S. et al. A MITE transposon insertion is associated with differential methylation at the maize flowering time QTL Vgt1. *G3* (Bethesda). **4**, 805-812 (2014).

45 Zhao, M. & Ma, J. Co-evolution of plant LTR-retrotransposons and their host genomes. *Protein Cell* **4**, 493-501 (2013).

46 Canaguier, A., et al. A new version of the grapevine reference genome assembly (12X.v2) and of its annotation (VCost.v3). *Genom Data*. 14, 56-62 (2017).

47 Cheng, C.Y., et al. Araport11: a complete reannotation of the Arabidopsis thaliana reference genome. Plant J. 89, 789-804 (2017).

48 Quinlan, A.R. & Hall. I.M. BEDTools: a flexible suite of utilities for comparing genomic features. *Bioinformatics*. **26**, 841-842 (2010).

49 Hao, Z. et al. RIdeogram: drawing SVG graphics to visualize and map genome-wide data on the idiograms. *PeerJ Comput Sci*. **6**, e251 (2020).

50 Ellinghaus, D. Kurtz, S. & Willhoeft, U. LTRharvest, an efficient and flexible software for de novo detection of LTR retrotransposons. *BMC bioinform* **9**, 18 (2008).

51 Xu, Z. & Wang, H. LTR_FINDER: an efficient tool for the prediction of full-length LTR retrotransposons. *Nucleic Acids Res.* **35**, W265-W268 (2007).
