# Supplementary material for: Large-scale discovery of non-conventional peptides in grape (Vitis vinifera L.) through peptidogenomics
Source: Hortic Res. 2022 May 2;9:uhac023. doi: 10.1093/hr/uhac023 (PMC9070638; doi:10.1093/hr/uhac023)
Supplement: Web_Material_uhac023 [file web_material_uhac023.zip › rebuttal letter.docx]

Dear Editor, Reviewers:

Thank you for your letter and for reviewers’ comments about our manuscript (HR-2021-176) entitled ‘Large-scale discovery of non-conventional peptides in grape (*Vitis vinifera* L.) through peptidogenomic’. All the comments are valuable and helpful for revising and improving our manuscript. We have studied the comments carefully and made corresponding revision. The point-by-point response to questions are listed as the following, and we hope that these answers and corrections will get your approval.

Yours sincerely,

Da-Long Guo

College of Horticulture and Plant Protection

Henan University of Science and Technology

No. 263, Kaiyuan Boulevard, Luolong District

Luoyang 471023, China

E-mail: guodalong@haust.edu.cn

The response to the reviewer’s comments are as following:

**Reviewer #1:**

Q1: In this study, mass spectrometry (MS) polypeptide genomics combined with a six-frame translation database was used to identify NCP in grapes on a large scale. 188 and 2021 non-redundant peptides were identified from the Grape URGI protein database, the Ensemble bl protein database and the custom peptide genome database, respectively. The results show that many previously thought untranslated regions, such as intergene, intron, upstream ORF, 5 UTR, 3 UTR and down-stream ORF, are translated and have potential functions in biological processes, which lays a foundation for studying the function of NCP. But the data of the form is not found in the submitted document, please upload and submit.

Response: Thank you very much for your positive comments for our manuscript. We have submitted the raw data to iProX (integrated proteome resources) (<https://www.iprox.org/>) with an accession number IPX0003909000 (https://www.iprox.cn//page/SCV017.html?query=IPX0003909000). The planed release time is 2022-12-29 00:00:00. Access prior to release date requires an account and password (account: peimaosong password: p12345). We have also supplemented the supporting data information in the manuscript, please see the detail in lines 474-480.

**Reviewer #2:**

Q1: How to determine whether non-coding regions harbored NCPs have been transcribed? It will be better if the RNA-Seq data from 'Kyoho' grape berries can be generated and investigated.

Response: We appreciate it very much for this good suggestion. We have the RNA-Seq data from ‘Kyoho’ grape berries and we have been back to investigate the overlap of NCPs locations with the PacBio SMRT Iso-seq data from ‘Kyoho’ grape berries which deposited in SRA (PRJNA790655) (<https://dataview.ncbi.nlm.nih.gov/object/PRJNA790655>), and the results revealed that 87 NCPs were overlapped with the RNA-seq reads, indicating that the NCPs were indeed transcribed. We added this information in Supplement Table S7, and randomly selected 10 NCPs for demonstrating using IGV. Please see details in figure 5 and corresponding description in lines 222-233. The related experimental methods were also supplemented in lines 445-450.

Q2. How to access the dataset (sequences and chromosomal locations) of NCPs and CPs identified in this study for a user?

Response: We have submitted the raw data to iProX (integrated proteome resources) (<https://www.iprox.org/>) with an accession number IPX0003909000 (https://www.iprox.cn//page/SCV017.html?query=IPX0003909000). The planed release time is 2022-12-29 00:00:00. Access prior to release date requires an account and password (account: peimaosong password: p12345). We have also supplemented the supporting data information in the manuscript, please see detail in lines 474-480.

Q3. Large-scale population genomic data for grapes have been available, and can be used to investigate the distribution of NCPs and CPs in selective regions during grape domestication and dissemination.

Response: Thanks a lot for your kind suggestion. We retrieved a large number of selective domestication regions based on the published literature, such as selective sweeps for domesticated table and wine grape, wild and domesticated grapes, berry edibility and stress resistance (Liang et al., 2020; Kui et al., 2020; Zou et al., 2021). When compared the location of NCPs to the selective regions during grape domestication and dissemination, 42 NCPs were screened to be overlapped with the selective regions (Figure 6b; Supplement Table S12). Please see details in lines 263-273.

Liang, Z., et al. Author Correction: Whole-genome resequencing of 472 Vitis accessions for grapevine diversity and demographic history analyses. *Nat Commun*. **11**, 2341 (2020).

Kui L, et al. Identification of Selective Sweeps in the Domesticated Table and Wine Grape (*Vitis vinifera* L.). *Front Plant Sci*. **11**, 572 (2020).

Zou C, et al. Multiple independent recombinations led to hermaphroditism in grapevine. *Proc Natl Acad Sci USA*. **118**, e2023548118 (2021).

Q4. Author use two paragraphs to describe the characteristics of NCPs (line 146-186) which is tedious. The author should combine the ‘Identification of CPs and NCPs in grape’ and ‘Distribution patterns of CPs and NCPs’ into one and make it concise.

Response: Thanks for your suggestion. We have combined the ‘Identification of CPs and NCPs in grape’ and ‘Distribution patterns of CPs and NCPs’ into ‘Identification and distribution mod of CPs and NCPs in grape’. Please see details in line 147.

Q5. In maize, compared with randomly selected genomic sequences with same distance distribution and number, SNPs significantly associated with plant traits appeared to be significantly enriched within the regions of NCPs (Shunxi Wang, 2020). This is a very interested result. The author should check your data and see if grape have similar pattern or opposite pattern which should be interesting and make your manuscript more comprehensive.

Response: Thank you for your nice suggestion. We investigated the relationship of NCPs and SNPs significantly associated with berry shape, berry weight, berry color, flower sex and intrinsic quality, etc (Guo et al., 2019; Liang et al., 2020; Kui et al., 2020). The results showed that 61 NCPs were overlapped with the 20-kb flanking regions of SNPs related to the investigated traits (Fig. 6c; Table S13). The SNPs associated with the trait were not significantly enriched within the regions of NCPs (p < 0.626, upper-tail test). These results were not similar with the NCPs in maize (Wang et al., 2020), which maybe indicated that the NCPs in different species have their unique characteristics. Please see details in lines 274-284.

Guo DL, et al. Genome-wide association study of berry-related traits in grape (*Vitis vinifera* L.) based on genotyping-by-sequencing markers. *Hortic Res*. **6**, 11 (2019).

Liang, Z., et al. Author Correction: Whole-genome resequencing of 472 Vitis accessions for grapevine diversity and demographic history analyses. *Nat Commun*. **11**, 2341 (2020).

Kui L, et al. Identification of Selective Sweeps in the Domesticated Table and Wine Grape (*Vitis vinifera* L.). *Front Plant Sci*. **11**, 572 (2020).

Q6. Author report that most of NCPs (94%) located in the QTLs and little in LTR-RT and selection regions but the subtitle is ‘NCPs located in regions with QTLs, LTR retrotransposons and domestication’ which is not an accurate and conclusive title should be revised to QTLs only because there is little NCPs in LTR-RT and selective regions.

Response: Thanks for your suggestion, we have revised the subtitle ‘NCPs located in regions with QTLs, LTR retrotransposons and domestication’ to ‘NCPs located in regions with QTLs’. Please see details in line 235.

Q7. If public data is used in this manuscript please use NCBI accession number instead of citing paper only.

Response: Thanks for your suggestion, the NCBI accession number of public data used in the manuscript was added in the manuscript. Such as the PacBio SMRT Iso-seq data from ‘Kyoho’ grape berries which deposited in SRA (PRJNA790655). Please see details in lines 445-447.

Q8. Grape and Arabidopsis genome papers should be cited in line 376

Response: Thanks for your suggestion, we have supplemented the Grape and *Arabidopsis* genome papers (numbered 46, 47 in reference list) in the manuscript. Please see details in lines 416、426、428、440 and 457.

Canaguier, A., et al. A new version of the grapevine reference genome assembly (12X.v2) and of its annotation (VCost.v3). Genom Data. 14, 56-62 (2017).

Cheng, C.Y., et al. Araport11: a complete reannotation of the Arabidopsis thaliana reference genome. Plant J. 89, 789-804 (2017).

Q9. All tools used in this manuscript should be cited: EMBOSS (line 379), bedtools (line 390), RIdeogram (line 397). Please double-check all software in your manuscript and make sure all the software is citied.

Response: Thanks for your suggestion, we have supplemented the references of EMBOSS-6.6.0, bedtools-v2.25.0, RIdeogram and IGV (numbered 16, 48, 49, 20, respectively in the reference list). All the software in our manuscript were cited. Please see details in line 142, 228, 419, 431, 437, 448, 450.

Rice, P., et al. EMBOSS: the European Molecular Biology Open Software Suite. *Trends Genet*. **16**, 276-277 (2000).

Thorvaldsdóttir, H., Robinson, J.T., & Mesirov, J.P. Integrative Genomics Viewer (IGV): high-performance genomics data visualization and exploration. *Brief Bioinform*. **14**, 178-192 (2013).

Quinlan, A.R. & Hall. I.M. BEDTools: a flexible suite of utilities for comparing genomic features. *Bioinformatics*. **26**, 841-842 (2010).

Hao, Z. et al. RIdeogram: drawing SVG graphics to visualize and map genome-wide data on the idiograms. *PeerJ Comput Sci*. **6**, e251 (2020).
